# A straightforward methodology to overcome solubility challenges for N-terminal cysteinyl peptide segments used in native chemical ligation†

**DOI:** 10.1039/d0sc06001a

**Published:** 2021-01-11

**Authors:** Skander A. Abboud, El hadji Cisse, Michel Doudeau, Hélène Bénédetti, Vincent Aucagne

**Affiliations:** Centre de Biophysique Moléculaire, CNRS UPR 4301 Rue Charles Sadron 45071 Orléans Cedex 2 France aucagne@cnrs-orleans.fr

## Abstract

One of the main limitations encountered during the chemical synthesis of proteins through native chemical ligation (NCL) is the limited solubility of some of the peptide segments. The most commonly used solution to overcome this problem is to derivatize the segment with a temporary solubilizing tag. Conveniently, the tag can be introduced on the thioester segment in such a way that it is removed concomitantly with the NCL reaction. We herein describe a generalization of this approach to N-terminal cysteinyl segment counterparts, using a straightforward synthetic approach that can be easily automated from commercially available building blocks, and applied it to a well-known problematic target, SUMO-2.

## Abstract 2

We herein describe a straightforward approach for the introduction of a solubilizing tag on N-terminal cysteinyl segments used in native chemical ligation-based protein chemical synthesis. Conveniently, the tag is removed during the ligation.

## Graphical abstract

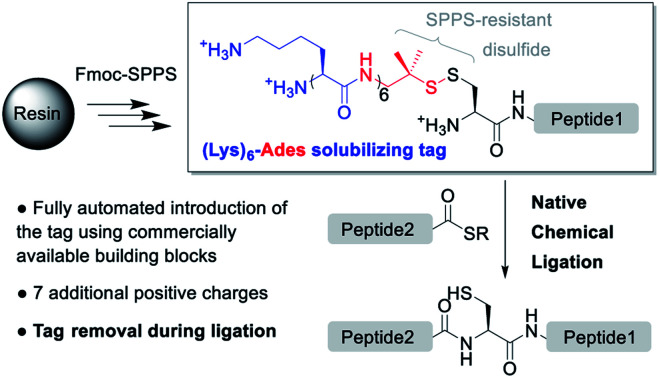

## Introduction

The advent of the native chemical ligation^1^ (NCL) reaction has revolutionized the field of chemical protein synthesis by offering a simple strategy to assemble unprotected peptide segments bearing mutually-reactive C-terminal thioesters and N-terminal cysteines with exquisite chemo- and regioselectivity. Twenty-six years after its discovery, NCL is still the gold standard reaction in the field. The continuous development of many related synthetic methodologies allowed to extend and simplify its applicability,^2^ and led to impressive applications in the total synthesis of functional proteins of more than 300 residues.^3^

One of the major current limitations of NCL-based protein synthesis is the low solubility or tendency to aggregate of some of the segments. If this is anticipated when synthesizing a very hydrophobic target such as a transmembrane protein, segments from soluble hydrophilic proteins frequently prove to be problematic, while being often very hard to predict. NCL is usually conducted under denaturing conditions, typically 6 M guanidinium chloride, and is tolerant to the addition of organic solvents^4^ and detergents,^5^ thus substantially minimizing these solubility/aggregation problems during the reaction itself. Nevertheless, the purification and characterization of the segments prior to NCL regularly remains a severe bottleneck. Many synthetic strategies were developed to overcome this critical problem. For example, modification of the segment through *N*-methylation of backbone amides^6^ or fusion with a hydrophilic peptide,^7^ can dramatically increase its solubility; however, these modifications will permanently remain in the synthesized protein. Traceless approaches include the use of acid-labile *N*-2-hydroxy-4-methoxybenzyl (Hmb) groups on backbone amides,^8^ or Ser/Thr *O*-acyl isopeptide^3*d*,*e*,9^ known to inhibit aggregation, but the most widely used strategy is the incorporation of a temporary hydrophilic “solubilizing tag”.^10^

Such temporary tags are generally composed of lysines or arginines that bear a cationic charge side chain, often as homo-oligomers, and can be introduced either on a backbone amide,^11^ the C-terminus^12^ of the segment or the side chain of an Asn,^13^ Asp,^13^ Cys,^12*c*,14,15^ Gln,^13,16^ Glu,^13,17*a*^ Lys^17^ or Thr^16^ residues.

**Scheme 1:**
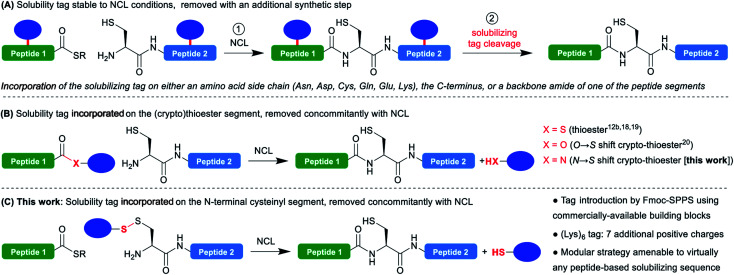
Synthetic strategies for solubilization of peptide segments used in NCL-based protein synthesis using temporary solubility tags (blue ovals) that are removed either after (A) or concomitantly (B and C) with the NCL reaction.

A large variety of linkers used to attach the tag to the peptide segment and designed to be cleaved in an additional step after the NCL (Scheme 1A) have been reported. Cleavage conditions include treatments with acids,^11,14*c*,*d*^ bases,^12*b*^ pH 4.5 buffer,^17*c*^ sodium nitrite,^12*c*,13^ nucleophiles,^17*b*,*d*^ transition metal catalysis,^12*c*,14*a*,*b*,15,17*a*^ UV irradiation^16^ and autoproteolysis.^12*a*^

An advantageous alternative is the introduction of the solubilizing tag on the C-terminal thioester moiety (Scheme 1B).^12*b*,18–20^ In this case, the tag is cleaved in the course of the NCL reaction without needing an additional synthetic step, after playing its solubilizing role during the purification, characterization and handling of the problematic segment. This approach was pioneered by Aimoto^18^ and Kent^19^ for Boc-SPPS-based thioester synthesis, and later extended by Tietze^20^ to Fmoc-SPPS using a β-mercaptoester precursor converted *in situ* into a thioester during NCL through an *O* → *S* acyl shift^21^ (a so-called peptide crypto-thioester^22^). If this one-pot NCL/tag cleavage approach is clearly not suited to extreme situations where the ligation product remains insoluble or prone to aggregation, it is expected to be widely applicable in the many cases when the low-solubility/aggregation behavior is associated with a single isolated segment of the protein. Indeed, the additional segment ligated to the problematic one can further play the role of solubility tag, strikingly demonstrated by the synthesis of fragments of transmembrane proteins using these techniques.^18,20^ However, the strategy is inherently limited to thioester segments and not suited for N-terminal cysteinyl counterparts.^23^

In a closely related context, Valiyaveetil^24^ proposed the idea to introduce a solubility tag linked through a disulfide to the N-terminal Cys of a cysteinyl segment synthesized by Boc-SPPS, the disulfide bond being cleaved in a first additional reduction step prior to NCL. In this case, the tag (Arg–Arg–Arg–Cys–NH_2_) was introduced in solution through air oxidation-mediated formation of a mixed disulfide with the crude segment. This resulted in a very low yield in the tagged segment due to both insolubility of the non-tagged segment and the non-directed formation of the disulfide leading to complex mixtures. Nevertheless, the authors succeeded in the highly demanding semi-synthesis of ion channels through expressed protein ligation^25^ (EPL) with a recombinant thioester, demonstrating the feasibility of the approach.

We thought that devising a straightforward methodology for the introduction of such a disulfide-linked solubilizing tag on N-terminal cysteinyl segments, and extending the concept of concomitant NCL/tag cleavage could be extremely valuable and generally applicable in chemical protein synthesis (Scheme 1C). Indeed, disulfides are readily cleaved under standard NCL conditions generally including reducing agents (*e.g.* TCEP) and a large excess of aromatic thiols like 4-mercaptophenylacetic acid (MPAA).^26^

We herein describe a straightforward methodology for the introduction of such a tag through Fmoc-SPPS, which can be easily automated on a standard peptide synthesizer, and exemplified the utility of this method through the synthesis of SUMO-2, a previously reported difficult target.^12*c*^

## Results and discussion

The disulfide-based linker bridging the N-terminal cysteinyl segment and the solubilizing tag is the cornerstone of the strategy. Ideally, this linker should be (1) introduced through an automation-friendly solid phase step using commercially available materials, (2) bear a primary aliphatic amine or a suitable precursor for further Fmoc-SPPS elongation of a hydrophilic peptide sequence and (3) be stable to the Fmoc-SPPS conditions, including elongation and TFA-based cleavage.

Considering these requirements, we reasoned that incorporation of the N-terminal cysteinyl residue through the coupling of Boc-Cys(Npys)-OH would be ideal. The Npys group^27^ (*S*-3-nitro-2-pyridinesulfenyl) is classically used for the directed formation of a mixed disulfide on an N-terminal cysteine either on solid support^28^ or in solution, through reaction with a thiol. The simplest solution for automated synthesis would be a reaction on solid support with an amino-thiol. We selected two commercially available candidates: cysteamine (2-amino-ethane-1-thiol, **1**), and 2-amino-1,1-dimethyl-ethane-1-thiol (**2**). Preliminary experiments with a model tripeptide (see ESI p. S5–S11†) showed that disulfide formation was quantitative through simple incubation in NMP for 1 h with an excess (10 equiv.) of **2** as its hydrochloride salt. We also demonstrated that the disulfide was stable to a long piperidine treatment mimicking the repeated Fmoc deprotection conditions needed for the SPPS elongation of a hydrophilic peptide tag. Contrastingly, reaction with **1** was much less clean, and the resulting disulfide was not stable to the piperidine treatment. These results are in accordance with the known higher stability of tertiary thiol disulfides derivatives of cysteine like *S*-S*t*Bu towards Fmoc-SPPS conditions as compared to simple non-hindered primary thiol disulfides.^29^

**Scheme 2:**
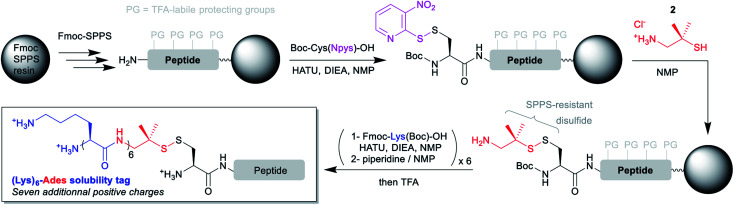
General synthetic strategies for the introduction of (Lys)_6_-Ades solubility tags on N-terminal cysteinyl segments using automated Fmoc-SPPS.

**Scheme 3:**
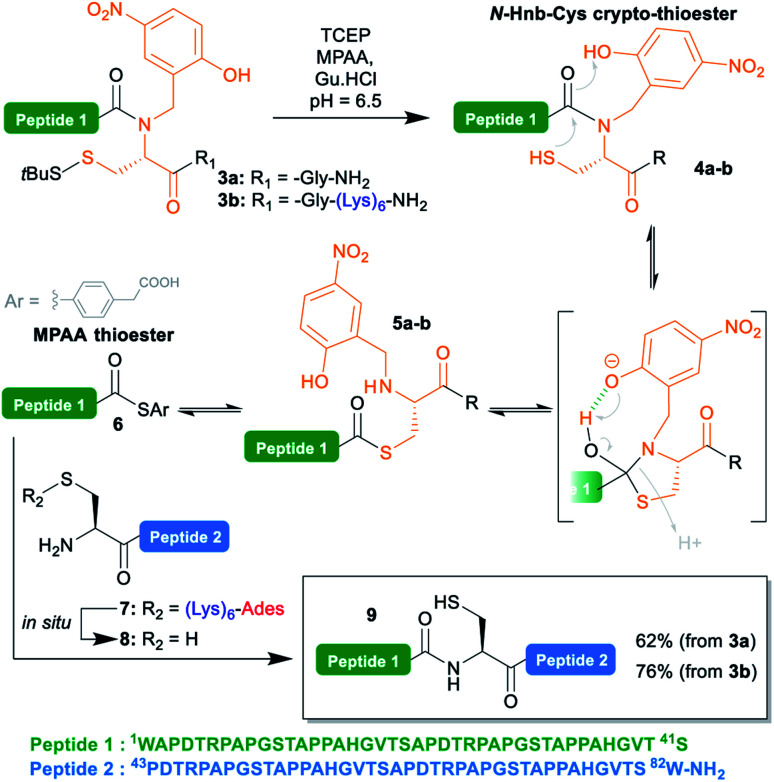
Native chemical ligation reaction showing the putative (Hnb)Cys self-catalyzed *N* → *S* acyl shift mechanism.

Having in hand a robust method for the solid phase introduction of the linker (referred hereafter as Ades, 2-amino-1,1-dimethylethyl-1-sulfanyl), we applied it to two different long peptides, followed by the automated introduction of a (Lys)_6_ hydrophilic tag through repeated couplings of Fmoc-Lys(Boc)-OH under standard conditions (Scheme 2). We started with a 41 amino acids (aa) model sequence devoid of any solubility problem, derived from the human mucin MUC1^30^ variable number tandem repeat (VNTR) region made of a duplicated 20 aa sequence (**7**, Scheme 3). Gratifyingly, **7** was obtained in excellent yield and purity without needing of any further optimization of the synthetic protocol.

To test the concomitant NCL/tag cleavage, we synthesized the 41 aa crypto-thioester segment counterpart **3a**, also derived from MUC1. This segment is equipped with an *N*-(2-hydroxy-4-nitrobenzyl)cysteine-based device (*N*-Hnb-Cys) capable of forming thioester *in situ* under NCL conditions (Scheme 3).^31^*N*-Hnb-Cys crypto-thioesters are straightforward to synthesize through automated Fmoc-SPPS and show fast ligation kinetics owing to internal catalysis by a judiciously placed phenol group (Scheme 3).^31*a*^

To our delight, the ligation of **3a** with **7** proceeded very cleanly, the (Lys)_6_-Ades tag being cleaved within seconds under the NCL conditions, giving the target 82 aa polypeptide **9** in a 62% isolated yield (ESI p. S18–S19).

Additionally, we took the opportunity of this work to demonstrate the applicability of the C-terminal thioester solubilization strategy to *N*-Hnb-Cys crypto-thioesters. Quite expectedly, introduction of a (Lys)_6_ tag was straightforward, and gave segment **3b** in good yields without needing any optimization. As for **3a**, NCL with **7** proceeded cleanly, and with comparable kinetics (ESI, Fig. S15†), giving **9** in an excellent 76% isolated yield (Scheme 3).

The cysteine residue in **9** was further desulfurized under classical conditions^32^ to give an alanine (ESI, p S20–S21) such as in the native MUC1 sequence.

Encouraged by these results, we then implemented this approach for the synthesis of the 93 aa SUMO-2. Small ubiquitin-related modifiers^33^ (SUMO) were first discovered in mammals in 1996.^34^ To dated, five SUMO isoforms have been identified in humans, SUMO-1, 2, 3, 4 and 5. SUMOylation is a post-translational modification (PTM) consisting in the covalent attachment of the C-terminus of SUMO proteins *via* an isopeptide bond to specific lysine residues in target proteins. An enzymatic cascade controls the attachment, involving activator (E1), conjugating (E2), and sometimes ligase (E3) enzymes. This PTM is reversible, through deSUMOylation by sentrin/SUMO-specific proteases (SENPs).^35^ In contrast with ubiquitin,^36,37^ only few examples of the chemical synthesis of SUMO proteins, their dimers and conjugates, have been reported.^5*d*,12*c*,38,39^ One illustration is the synthesis by Brik^12*c*^ of SUMO-2-diubiquitin hybrid chains. In this work, the authors reported the low solubility, tendency to aggregation and unusual HPLC behavior of an N-terminal cysteinyl SUMO-2[46–93]^40^ segment. To circumvent this issue, they developed an elegant C-terminal solubilizing tag in which a 3,4-diaminobenzoic acid (Dbz) linker^41^ was employed to attach a poly-Arg tag to the C-terminus of this segment. We thought that it could be interesting to challenge our (Lys)_6_-Ades methodology with this benchmark target.

**Scheme 4:**
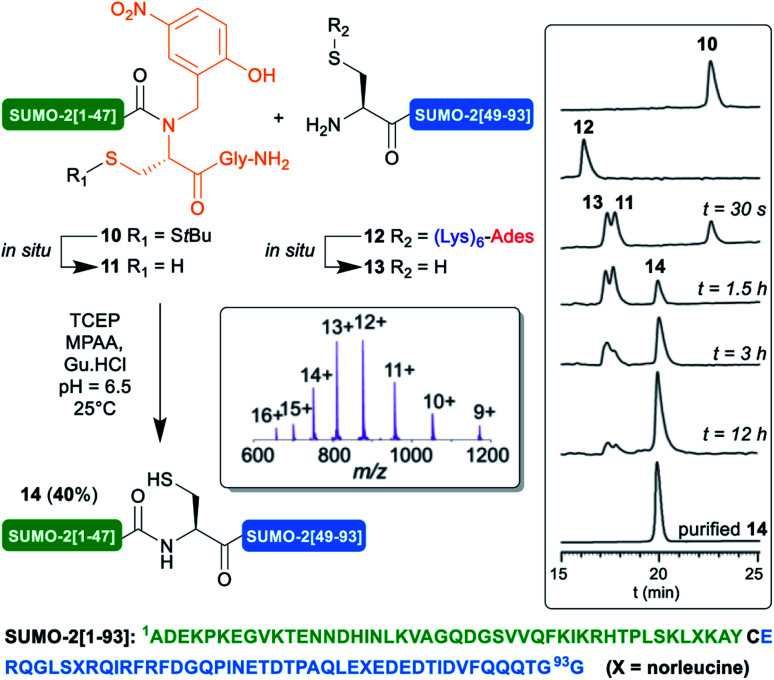
NCL-based SUMO-2 synthesis using the (Lys)_6_Ades methodology.

We applied our synthetic protocol to (Lys)_6_-Ades-SUMO-2[48–93] (**12**),^42^ which gave a clean and soluble crude product that was purified by standard HPLC. As anticipated from Brik's report, in sharp contrast with **12**, the non-tagged version exhibited anomalous HPLC behavior (ESI, S11, Fig. S11†) and the crude peptide was barely soluble in HPLC solvents: 0.05 mg mL^−1^ in 8 : 2 : 0.01 H_2_O/MeCN/TFA, thus further validating the choice of this target for this work. Gratifyingly, NCL of **12** with SUMO-2[1–47] *N*-Hnb-Cys crypto thioester **10** proceeded very cleanly as shown in the analytical HPLC monitoring^43^ of the reaction (Scheme 4), giving the expected SUMO-2[1–93] **14** in a good 40% isolated yield. As observed with the MUC1-derived model segments, the HPLC chromatograms show nearly instantaneous conversion of **12** into non-tagged **13**, concomitantly with the slightly slower conversion of *S*-S*t*Bu-protected dormant *N*-Hnb-Cys crypto-thioester **10** into an active form **11**.^44^

Note that in accordance with Melnyk's findings,^45^ 15% of a side product showing a molecular mass of [M − 18] *m*/*z* relative to the desired product **14** was observed when performing the reaction at 37 °C for a prolonged time and was attributed to aspartimide formation at one of the Asp–Gly sites. Lowering the temperature to 25 °C nearly abolished this side reaction (ESI p. S22–S25).

Finally, in order to further characterize the synthesized SUMO-2 **14** from a biochemical point of view, it was folded by simple solubilization into a neutral buffer as described.^39*b*,*f*,46^ Its three-dimensional structure was evaluated by circular dichroism, showing a spectrum essentially identical with the previously reported ones^46^ for recombinant and synthetic SUMO-2 (ESI Fig. S21†).

**Fig. 1:**
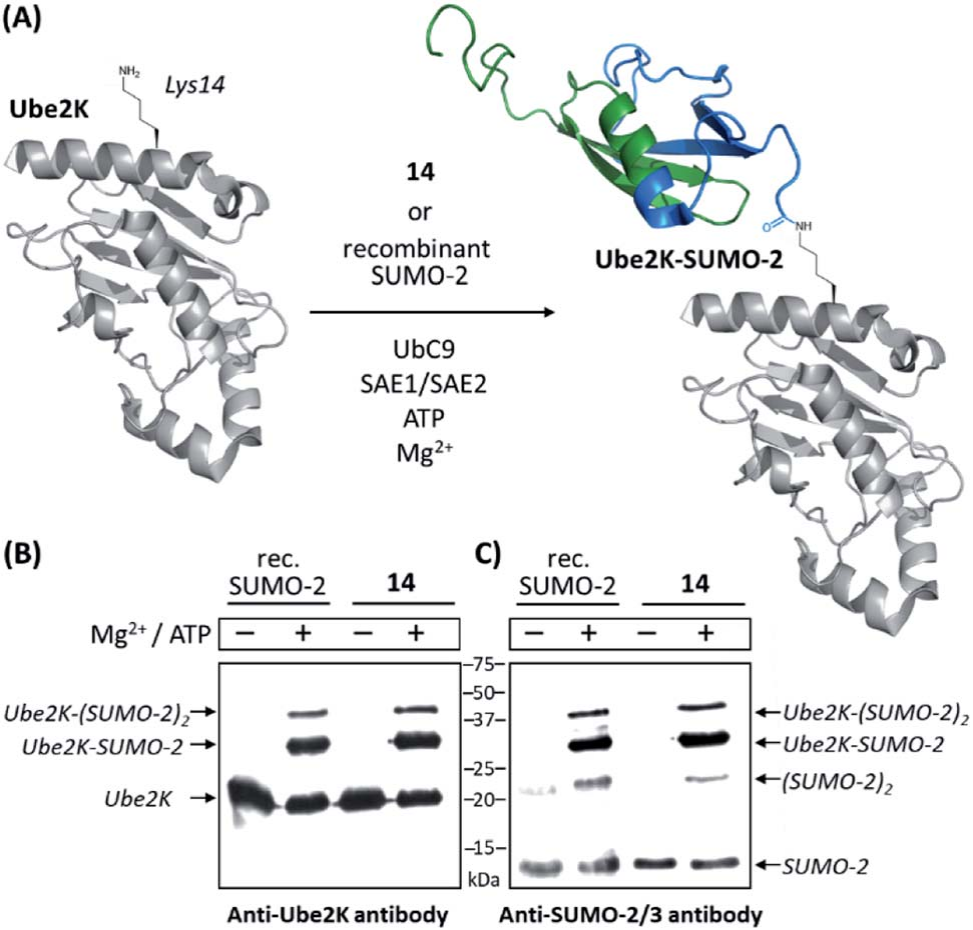
*In vitro* SUMOylation of Ube2K with **14** and a recombinant SUMO-2 used as a control. (A) Schematic representation of the reaction (cartoons based on NMR and X-ray structures of SUMO-2 and Ube2K, PDB ID: 2BEP and 2N1W, respectively); (B) Western blot analysis using an anti-Ube2K antibody; (C) Western blot analysis using an anti-SUMO-2/3 antibody.

We then compared **14** to a commercially available recombinant version of SUMO-2 for its ability to act as a substrate of the SUMO E2 conjugating enzyme Ubc9 (ref. 47) and a SUMO E1 activating enzyme (namely a heterodimeric complex of SAE1 and SAE2 proteins)^48^ for conjugation to the ubiquitin-conjugating enzyme Ube2K (also known as E2-25K and Huntington Interacting Protein 2). This target is reported to be among the best *in vitro* substrates for Ubc9-dependent SUMOylation known thus far, although its SUMOylated lysine residue (Lys14) is not surrounded by a consensus SUMOylation motif.^49^ Using anti-SUMO-2/3 and anti-Ube2K antibodies, Western-blot analyses first showed that synthetic SUMO-2 is recognized as efficiently as the recombinant one. The appearance of an intense band recognized by both antibodies and migrating at the expected molecular mass of a SUMO-modified Ube2K demonstrated the successful SUMOylation of the acceptor protein in both cases. Weaker bands likely corresponding to di-SUMOylated Ube2K and di-SUMO-2 were also observed, consistent with the presence of a SUMOylable lysine in SUMO-2 (Lys11). As expected, no reactions occurred in the absence of ATP and Mg^2+^, required cofactors for the E1 enzyme. Altogether, these experiments demonstrate the fully functional nature of synthesized **14** (Fig. 1 and ESI p. S27–S29).

## Conclusions

Poor solubility and aggregation of peptide segments are main bottlenecks for the chemical synthesis of proteins using native chemical ligation. Numerous strategies for the solubilization of problematic segments through temporary modification have been developed such as the introduction of solubilizing tags, but often require complex synthetic strategies, in-house synthesis of building blocks or extra steps to generate the native protein sequence after the NCL-based assembly. In this work, we have introduced a straightforward methodology for the temporary solubilization of N-terminal cysteinyl segments, based on the introduction of an oligolysine tag through a disulfide linkage with the N-terminal cysteine residue. This (Lys)_6_-Ades tag is easily incorporated in the target segment through automated solid-phase synthesis using commercially available building blocks, is stable during handling, purification and storage of the segment, while being cleaved within seconds under NCL conditions^50^ to generate *in situ* the reactive free cysteine. We exemplified the broad potential of this method through the NCL-based synthesis of a model polypeptide derived from the human mucin MUC1, in addition to a well-known difficult small protein target, SUMO-2. Due to its overall simplicity and efficiency, we believe that this strategy will advantageously complement existing methodologies in the synthesis of other challenging proteins.

## Conflicts of interest

There are no conflicts to declare.

## Supplementary Material

SC-012-D0SC06001A-s001
