# Supplementary material for: A straightforward methodology to overcome solubility challenges for N-terminal cysteinyl peptide segments used in native chemical ligation
Source: Chem Sci. 2021 Jan 11;12(9):3194–201. doi: 10.1039/d0sc06001a (PMC8179351; doi:10.1039/d0sc06001a)
Supplement: SC-012-D0SC06001A-s001 [file SC-012-D0SC06001A-s001.pdf]

Electronic Supplementary Material (ESI) for Chemical Science.
This journal is © The Royal Society of Chemistry 2021

A straightforward methodology to overcome solubility challenges for *N*-terminal cysteinyl segments used for native chemical ligation.

Skander A. Abboud, El hadji Cisse, Michel Doudeau, Hélène Bénédetti, Vincent Aucagne*

Centre de Biophysique Moléculaire, CNRS UPR 4301, Rue Charles Sadron 45071 Orléans cedex 2, France. E-mail: aucagne@cnrs-orleans.fr

Supporting Information

Table of contents:

1- General information......2

2- General procedures for solid phase peptide synthesis......2

3- Optimization of the disulfide linker on a model tripeptide......5
- **3-1-** Synthesis of Boc-Cys(Npys)-peptidyl resin **S3**......5
- **3-2-** Reaction of 2-aminoethane-1-thiol **1** with **S3** to give **S4**......6
- **3-3-** Reaction of 2-amino-1,1-dimethyl-ethane-1-thiol **2** with **S3** to give **S5**......7
- **3-4-** Stability of disulfide-containing peptidyl resins **S4** and **S5** towards piperidine treatment......8
- **3-5-** Optimization of the reaction of **2** with Boc-Cys(Npys)-peptidyl resin **S3**......10

4- Synthesis and purification of MUC1-derived peptide segments......11
- **4-1-** H-Trp-$(MUC1)_2$-(Hnb)Cys(S*t*Bu)-Gly-NH_2 crypto-thioester (**3a**)......11
- **4-2-** H-Cys(K_6-Ades)-$(MUC1)_2$-Trp-NH_2 cysteinyl peptide (**7**)......12
- **4-3-** H-Trp-$(MUC1)_2$-(Hnb)Cys(S*t*Bu)-Gly-$(Lys)_6$-NH_2 crypto-thioester (**3b**)......13

5- Synthesis and purification of SUMO-2-derived peptide segments......14
- **5-1-** SUMO-2[1-46]-(Hnb)Cys(S*t*Bu)-Gly-NH_2 crypto-thioester (**10**)......14
- **5-2-** SUMO-2[47-93] cysteinyl peptide (**S11**)......15
- **5-3-** Cys(K_6-Ades)-SUMO-2[48-93] cysteinyl peptide (**12**)......16

6- Native chemical ligations......18
- **6-1-** NCL between **3a** and **7** to give **9**......18
- **6-2-** NCL between **3b** and **7** to give **9**......19
- **6-3-** Desulfurization of **9**......20
- **6-4-** NCL between **10** and **12** to give SUMO-2[1-93] (**14**)......22

7- Functional and structural characterization of SUMO-2[1-93] (14)......26
- **8-1-** Circular dichroism......26
- **8-2-** Enzymatic conjugation to an acceptor protein......27

1- General information

All reagents and solvents were used without further purification. Protected amino acids were purchased from Gyros Protein Technology (Uppsala, Sweden). 1-Amino-2-methyl-2-propanethiol hydrochloride (**2**.HCl) was purchased from Key organics (Camelford, UK). DIEA was purchased from Carlo Erba (Val-de-Reuil, France). Rink amide ChemMatrix resin was purchased from Biotage (Uppsala, Sweden). Peptide synthesis grade DMF was obtained from VWR (Fontenay-sous-Bois, France). Fmoc-Cys(NPys)-OH was purchased from BACHEM (Bubendorf, Switzerland). All other chemicals were from Sigma Aldrich (St-Quentin-Fallavier, France) and solvents from Carlo Erba. Ultrapure water was obtained using a Milli-Q water system from Millipore (Molsheim, France). Polypropylene syringes fitted with polypropylene frits were obtained from Torviq (Niles, MI, USA) and were equipped with PTFE stopcocks bought from Biotage. HPLC analyses were carried out on a Chromaster system equipped with a 5160 pump, a 5430 diode array detector and a 5260 auto sampler and semi-preparative purifications were carried out on a LaChromElite system equipped with a Hitachi L-2130 pump, a Hitachi L-2455 diode array detector and a Hitachi L-2200 auto sampler. Chromolith High Resolution RP-18e (150 Å, 10 × 4.6 mm, 3 mL/min flow rate) columns were used for analysis, Nucleosil C18 (300 Å, 5 µm, 250 × 10 mm, 3 mL/min flow rate) and Jupiter C4 (300 Å, 5 µm, 250 × 10 mm, 3 mL/min flow rate) for purification. Solvents A and B are 0.1% TFA in H_2O and 0.1% TFA in MeCN, respectively. Each gradient was followed by a washing step to elute any compound not eluted during the gradient (up to 95% B/A over 0.5 min, then isocratic 95% B/A for 0.5 min for the HR Chromolith). LC-MS analyses were carried out on an Agilent 1260 Infinity HPLC system, coupled with an Agilent 6120 mass spectrometer (ESI + mode), and fitted with an Aeris Widepore XB-C18 2 (3.6 µm, 150 × 2.1 mm, 0.5 mL/min flow rate, 60°C) column. The reported *m*/*z* values correspond to the monoisotopic ions if not specified otherwise. Solvents A' and B' were 0.1% formic acid in H_2O and 0.1% formic acid in MeCN, respectively. Gradient: 3% B'/A' for 0.6 min, then 3 to 50% B'/A' over 10.8 min. Low resolution MS of pure compounds were obtained using this system. When needed, the multiply-charged envelope was deconvoluted using the charge deconvolution tool in Agilent OpenLab CDS ChemStation software to obtain the average [M] value. High resolution ESI-MS analyses were performed on a maXis™ ultra-high-resolution Q-TOF mass spectrometer (Bruker Daltonics, Bremen, Germany), using the positive mode. The multiply-charged envelope was deconvoluted using the charge deconvolution algorithm in Bruker Data Analysis 4.1 software to obtain the monoisotopic [M] value.

For yield calculations purposes, the quantities of purified peptides were determined by weight, taking into account a molecular weight including trifluoroacetate counter-ions (one per Arg, His, Lys and N-terminal amine of the peptide sequence) but not water content.

2- General procedures for solid phase peptide synthesis

Protocol PS1- Peptide elongation: Automated Fmoc-based solid phase peptide syntheses (SPPS) were carried out on a Prelude synthesizer from Protein technologies. Manual couplings were performed on polypropylene syringes fitted with polypropylene frits using rotation stirring. The side-chain protecting groups used were Arg(Pbf), Asp(O*t*Bu), Cys(Npys), Cys(Trt), Cys(S*t*Bu), Glu(O*t*Bu), Gln(Trt), His(Trt), Lys(Boc), Ser(*t*Bu), Thr(*t*Bu), and Tyr(*t*Bu). Syntheses were performed on a 0.025 mmol-per-reactor scale. Protected amino acids (0.25mmol, 10 equiv.) were coupled using HATU (90 mg, 0.238 mmol, 9.5 equiv.) and DIEA (87 µL, 0.5 mmol, 20 equiv.) in NMP (3 mL) for 30 min. Coupling on *N*-Hnb-cysteine secondary amine were performed through three successive couplings for 2 h. Capping of possible unreacted amine groups was achieved by treatment with acetic anhydride (143 µL, 1.51 mmol, 60 equiv.), DIEA (68 µL, 0.39 mmol, 15.5 equiv.) and HOBt (6 mg, 0.044 mmol, 1.8 equiv.) in NMP (3 mL)

for 7 min (4 x 7 min in the case of *N*-Hnb-cysteine secondary amine). Fmoc group was deprotected by three successive treatments with 20% piperidine in NMP (3 mL) for 3 min.

Protocol PS2-Reductive amination: introduction of the hydroxyl nitrobenzyl group (Hnb): 25 µmol of H-Cys(S*t*Bu)-Gly-Rink-resin (1a-c) was washed with 1:1 DMF/MeOH (4 x 3 mL, 30 s). 2-Hydroxy-5-nitrobenzaldehyde (42 mg, 10 equiv.) in 2 mL 44.5:44.5:1 DMF/MeOH/AcOH (125 mM aldehyde concentration) was then added, and the reactor was stirred for 5 min. The reactor was drained and the resin was washed with 1:1 DMF/MeOH (3 x 3 mL, 5 s) then DMF (3 x 3 mL, 5 s). Without delay, a fresh solution of sodium borohydride (19 mg, 20 equiv.) in 2 mL DMF (250 mM borohydride concentration) was added and the reactor was stirred for 20 min. The reactor was drained and the resin was washed with DMF (4 × 3 mL, 30 s), 20% *v*/*v* piperidine in NMP (3 × 3 mL, 3 min), NMP (3 × 3 mL, 30 s), dichloromethane (3 × 5 mL, 30 s) and NMP (3 × 3 mL, 30 s).

Protocol PS3-Boc-Cys(Ades) introduction: 25 µmol of peptidyl resin was washed with 3 mL of NMP. A solution of Boc-Cys(Npys)-OH (93.5 mg, 10 equiv.), HATU (90.3 mg, 9.5equiv.) and DIEA (87 µL, 20 equiv.) in 2.5 ml of NMP (100 mM Boc-Cys(Npys)-OH) was added to the resin and the reactor was stirred for 4 hours. The reactor was drained and the resin was washed with DCM (3 × 3 mL, 30 s) and NMP (3 × 3 mL, 30 s). A solution of 2-amino-1,1-dimethyl-propane-1-thiol hydrochloride **2** (35.5 mg, 10 equiv.) in 2.5 ml of NMP (100 mM thiol) was then added to the resin and the reactor was stirred for 5 min. The reactor was drained off and the resin was washed with DCM (3 × 3 mL, 30 s) and NMP (3 × 3 mL, 30 s).

Protocol PS4-Peptide cleavage: The crude peptide was deprotected and cleaved from the resin through a treatment with TFA/H_2O/*i*Pr_3SiH/phenol, 88:5:2:5 for 2 h, and the peptide was precipitated by dilution into an ice-cold diethyl ether/petroleum ether 1:1 mixture, recovered by centrifugation and washed twice with diethyl ether.

Protocol PS5-Small peptides cleavage (less than 4 residues): Small peptides were cleaved from the resin through a treatment with TFA/H_2O/*i*Pr_3SiH, 93:5:2 for 30 min following by concentration under vacuum.

Protocol PS6 - Procedure for selective Hnb ester cleavage to allow UV titration of Fmoc deprotection: As a consequence of the formation of variable amount (5-90 %) of *O*-acylated Hnb during each coupling, this ester being cleaved upon piperidine treatment during Fmoc deprotection, standard UV titration of the fluorenylmethyl-piperidine adduct after Fmoc deprotection is useless unless using a prior treatment for selective ester cleavage before piperidine treatment. Ester cleavage mixture was prepared as follows: 1.25 g (1.80 mmol) of $NH_2OH{\cdot}HCl$ and 0.92 g (1.35 mmol) of imidazole were suspended in 5 mL of NMP and the mixture was sonicated until complete dissolution. This solution can be stored for few months at -20 °C. 5 volumes of this this solution is diluted with 1 volume of DCM prior to utilization, and the resin is treated with the resulting mixture for 3 × 20 min for quantitative ester cleavage. The fluorenylmethyl-piperidine adduct is quantified by UV spectroscopy at λ = 301 nm (ε = 7800 L mol^{-1} cm^{-1}) in order to evaluate the Fmoc SPPS elongation yield of crypto-thioester peptides.

Protocol PS7-General procedure for native chemical ligation:

Preparation of the NCL buffer (100 mM MPAA, 50 mM TCEP, 6 M guanidinium chloride, 200 mM sodium phosphate, pH = 6.5) :

16.8 mg 4-mercaptophenylacetic acid (MPAA, 0.18 mmol), 14.3 mg tris-carboxyethylphosphine hydrochloride (TECP, 0.057 mmol) and 10.3 mg of dry NaOH powder (0.26 mmol) were weighted into a vial, which was sealed with a septum and purged with an argon flow.

A 200 mM disodium hydrogen phosphate, 6 M guanidinium chloride aqueous solution was prepared by dissolving 356 mg disodium hydrogen phosphate dihydrate (2 mmol) and 5.73 g guanidinium chloride in water (10 mL final volume). This solution can be kept at 4°C for several months. Just before use, this solution was thoroughly deoxygenated through successive vacuum/argon cycles.

Under argon, 1 mL of this solution was added to the MPAA/TCEP/NaOH vial, followed by sonication upon complete dissolution to give the NCL buffer which was immediately used.

<u>NCL reaction</u>: The cysteinyl peptide and crypto-thioester were weighted in a centrifuge tube, which was sealed with a septum and purged with an argon flow. The volume of the NCL buffer appropriate to reach the desired final peptide segments concentration was added under argon, the reaction vessel was sealed with Parafilm, and the resulting yellow solution was allow to stir at 25 °C or 37 °C. The reaction was quenched by dilution into 20 volumes of an 80:20:0.1 water/MeCN/TFA mixture.

3- Optimization of the disulfide linker on a model tripeptide

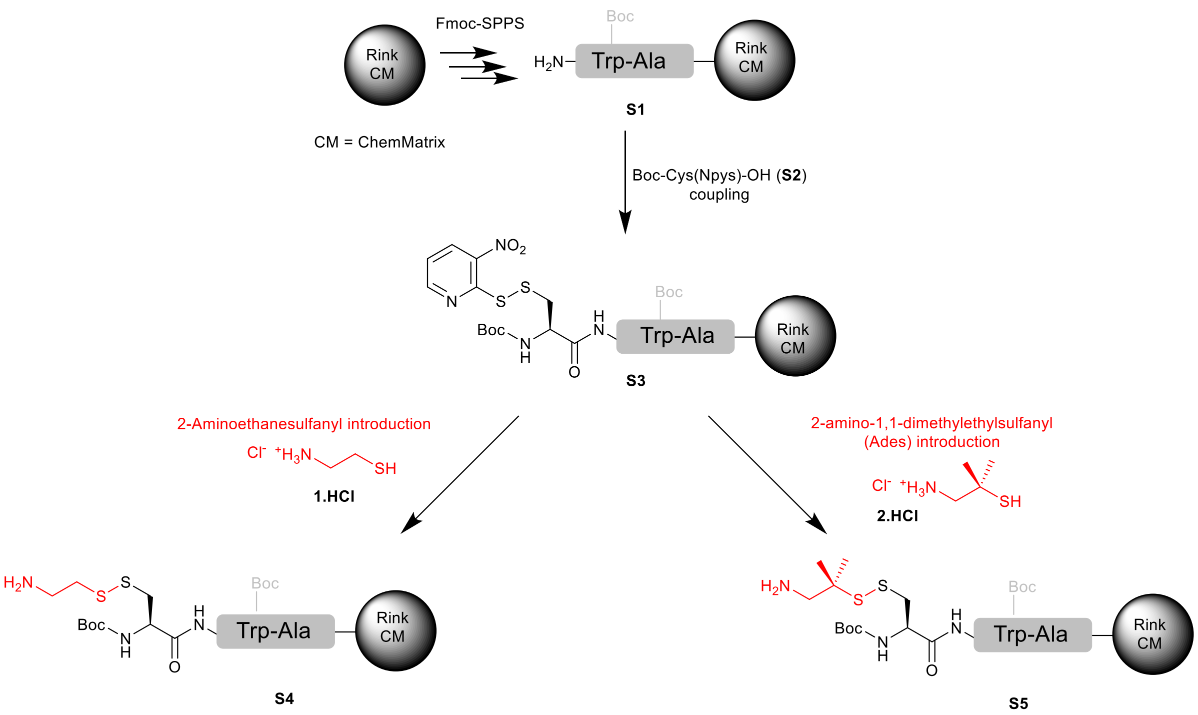

3-1- Synthesis of Boc-Cys(Npys)-peptidyl resin S3

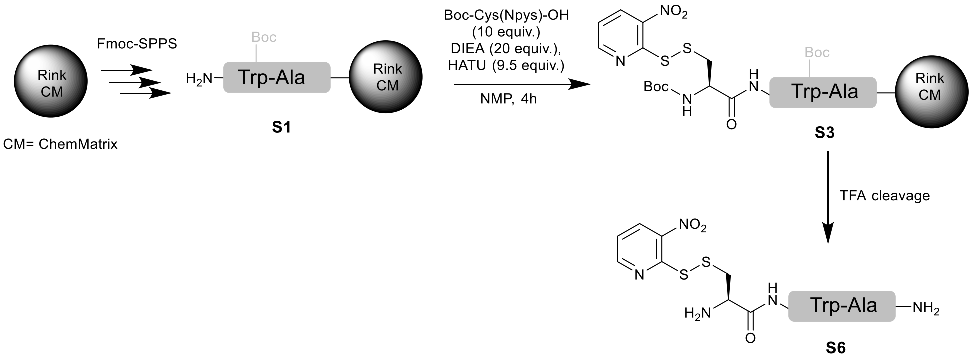

Peptidyl resin **S3** was obtained through manual SPPS (protocol PS1) starting from Rink-ChemMatrix resin (0.4 mmol/g). The coupling of Boc-Cys(Npys)-OH was performed as described in protocol PS3. A small amount of the peptidyl resin was cleaved for analytical purpose to afford tripeptide **S6** (protocol PS5 small peptides cleavage).

- H-Cys(Npys)-Trp-Ala-NH_2 (**S6**):

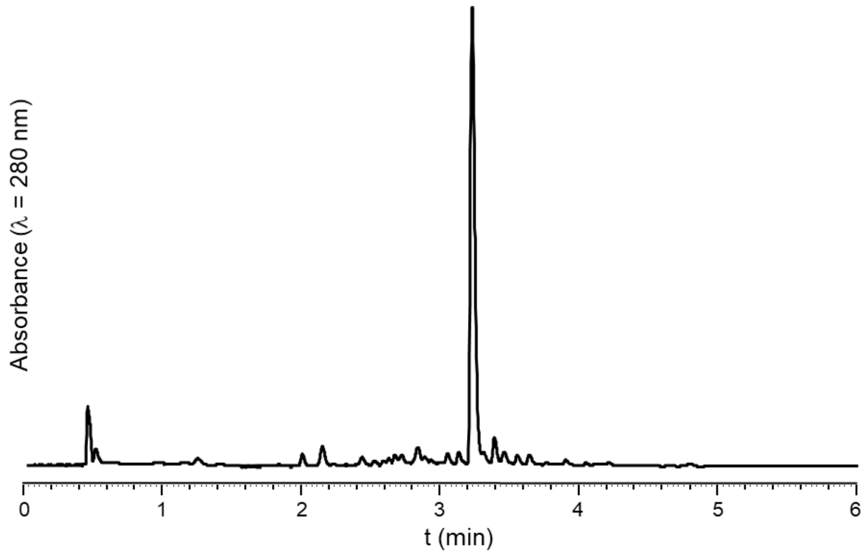

Supplementary figure S1: HPLC trace of crude **S6**

ESI-MS (*m/z*): [M] calcd. for $C_{22}H_{25}N_7O_5S_2$: 531.1, found: 531.1

HPLC analysis: t_R = 3.22 min (Chromolith, gradient: 5-50% B/A over 5 min)

3-2- Reaction of cysteamine hydrochloride 1.HCl with S3 to give S4

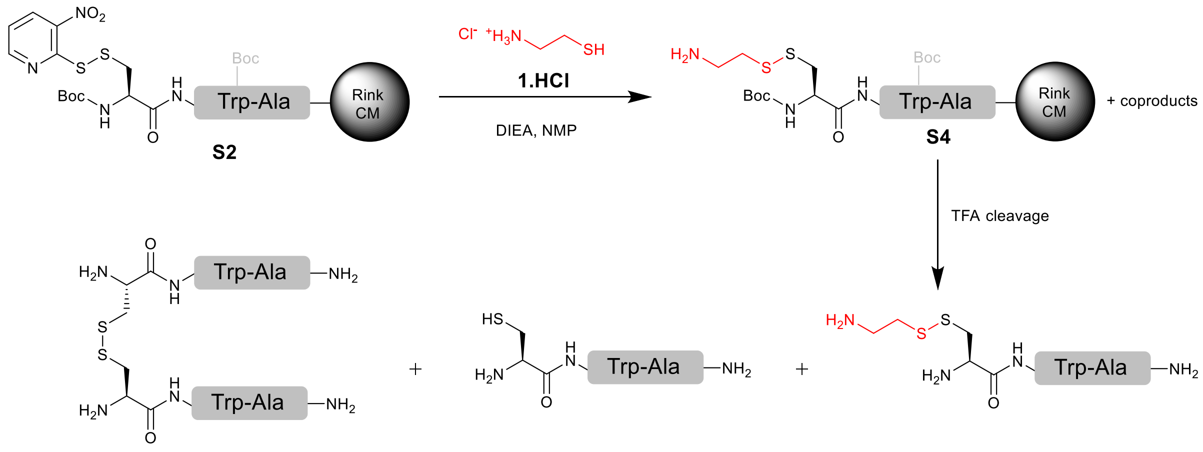

10 µmol of peptidyl resin **S2** was washed with 3 mL of NMP. A solution of 2-aminoethanethiol hydrochloride (8 mg, 10 equiv.) in 1 ml of NMP (100 mM thiol concentration) was added to the resin. Then DIEA (37.8 µL, 20 equiv.) was added and the reactor was stirred for 5 min. The reactor was drained and the resin was washed with DCM (3 × 3 mL, 30 s) and NMP (3 × 3 mL, 30 s). A small amount of the peptidyl resin was cleaved for analytical purpose affording crude **S7** (protocol PS5 small peptides cleavage).

- H-Cys($SCH_2CH_2NH_2$)-Trp-Ala-NH_2 (**S7**):

ESI-MS (*m/z*): [M] calcd. for $C_{19}H_{28}N_6O_3S_2$: 452.2, found: 452.1

HPLC analysis: t_R = 2.22 min (Chromolith, gradient: 5-50% B/A over 5 min)

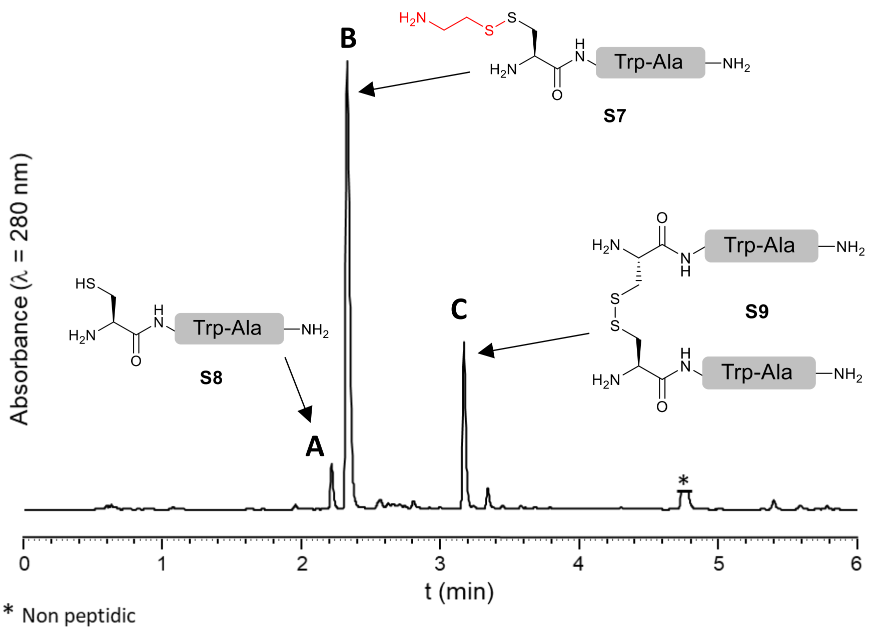

Peak (t_R (min))	[M] (*m/z*) calcd.	[M] (*m/z*) found	Attributed to
A (2.36)	377.1	377.2	**S8**
B (2.22)	452.2	452.1	**S7**
C (3.20)	752.3	752.2	**S9**
* (5.00)	No MS signal		Non peptidic compound

Supplementary figure S2: HPLC trace and MS characterisation of crude **S7**

3-3- Reaction of 2-amino-1,1-dimethyl-ethane-1-thiol hydrochloride with S2 to give S5

(Non-optimized conditions)

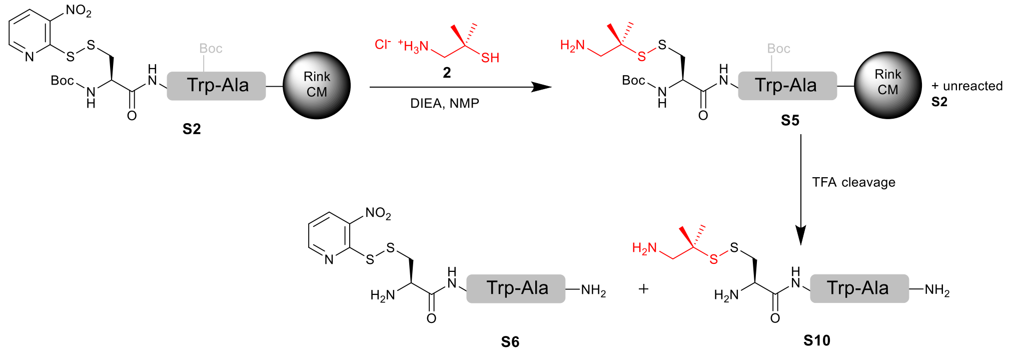

10 µmol of peptidyl resin **S3** was washed with 3 mL of NMP. A solution of 2-amino-1,1-dimethyl-propane-1-thiol hydrochloride **2** (14 mg, 10 equiv.) in 1 ml of NMP (100 mM thiol concentration) was added to the resin. Then DIEA (35 µL, 20 equiv.) was added and the reactor was stirred for 5 min. The reactor was drained off and the resin was washed with DCM (3 × 3 mL, 30 s) and NMP (3 × 3 mL, 30 s). A small amount of the peptidyl resin was cleaved for analytical purpose affording **S10** (protocol PS5 small peptides cleavage).

- H-Cys(H-Ades)-Trp-Ala-NH_2 (**S10**):

ESI-MS (*m/z*): [M] calcd. for $C_{21}H_{32}N_6O_3S_2$: 480.2, found: 480.2

HPLC analysis: t_R = 2.49 min (Chromolith, gradient: 5-50% B/A over 5 min)

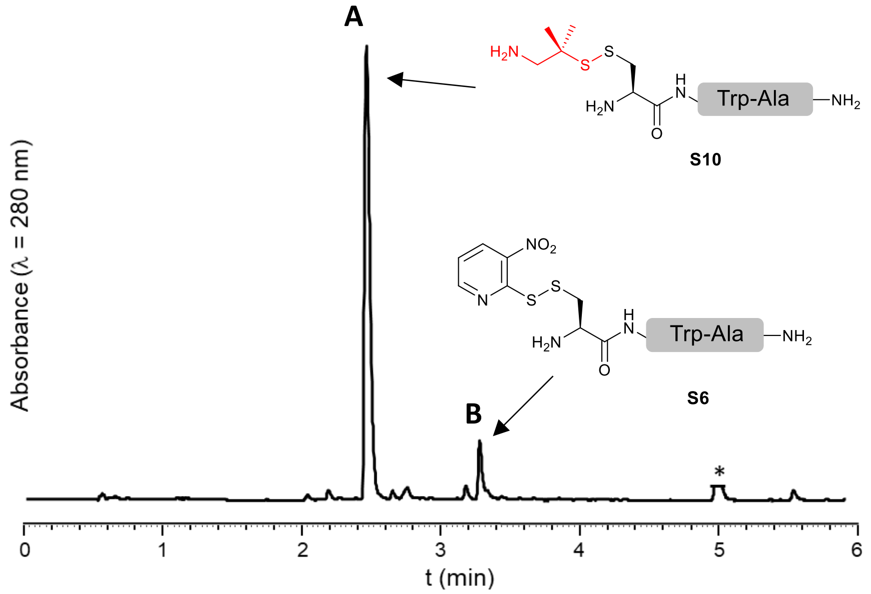

*: non-peptidic contaminant

Peak (t_R (min))	[M] (*m/z*) calcd.	[M] (*m/z*) found	Attributed to
A (2.49)	480.2	480.2	**S10**
B (3.31)	377.1	377.2	**S6**
* (5.00)	No MS signal		Non peptidic compound

Supplementary figure S3: HPLC trace and MS characterisation of crude **S10** obtained under non-optimized conditions

3-4- Stability of disulfide-containing peptidyl resins S4 and S5 towards piperidine treatment

Resins were washed with 3 mL of NMP. A solution of 20 % *v*/*v* piperidine in NMP was added and the reactor was stirred for 1 h. The reactor was drained off and the resin was washed with DCM (3 × 3 mL, 30 s) and NMP (3 × 3 mL, 30 s). A small amount of the peptidyl resin was cleaved for analytical purpose (protocol PS5 small peptides cleavage).

- **Peptidyl resin S4**

Complete degradation of **S4** was noticed upon treatment with piperidine, as assessed by TFA cleavage followed by analytical HPLC.

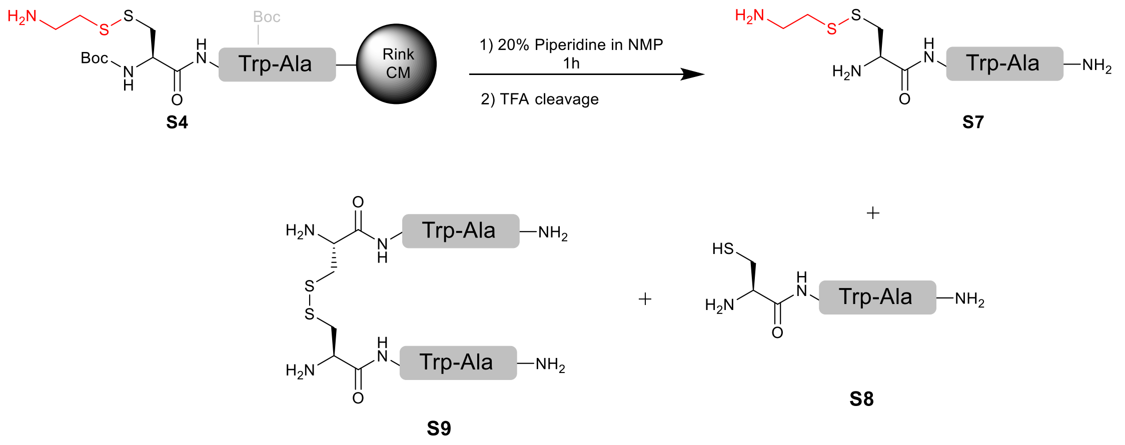

- Supplementary table S1: Rates of the products formed after piperidine treatment of peptidyl resin **S4** followed by TFA cleavage.

Entry	Piperidine treatment	S7 (%)[a]	S8 (%)[a]	S9 (%)[a]
1	No	79	10	11
2	Yes	-	58	42

[a]: Relative rates determined by HPLC peak integration at λ= 280 nm, taking into account the predicted molar absorption coefficient of Trp and disulfide at 280 nm: ε_{Trp} = 5500 L.mol^{-1}.cm^{-1}; ε_{SS} = 125 L.mol^{-1}.cm^{-1}. [1]

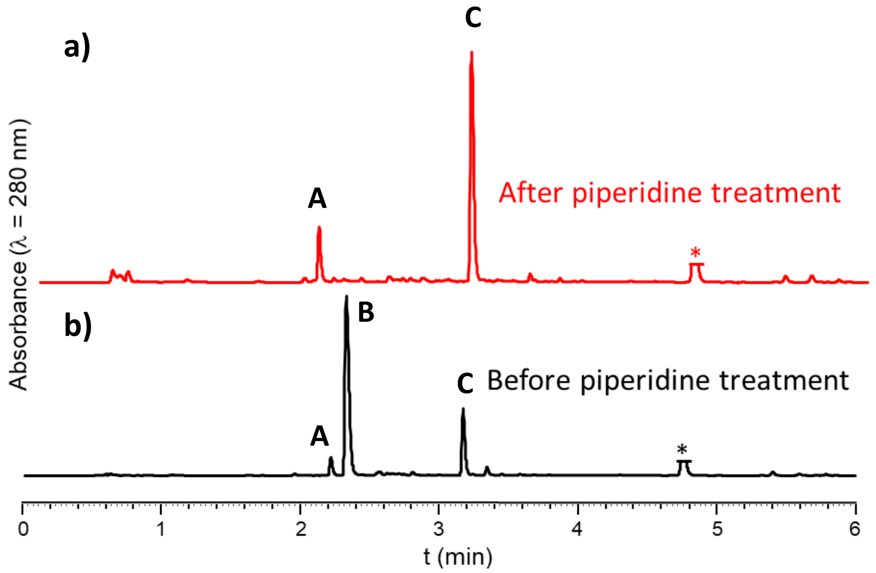

*: non-peptidic contaminant

Supplementary figure S4: HPLC trace (Chromolith, gradient: 5-50% B/A over 5 min) of TFA-cleaved peptidyl resin **S4** with (a) and without (b) prior piperidine treatment

- **Peptidyl resin S5**

No degradation of **S5** was noticed upon treatment with piperidine, as assessed by TFA cleavage followed by analytical HPLC, showing no evolution of the major peak, corresponding to H-Cys(H-Ades)-Trp-Ala-NH_2 (**S10**).

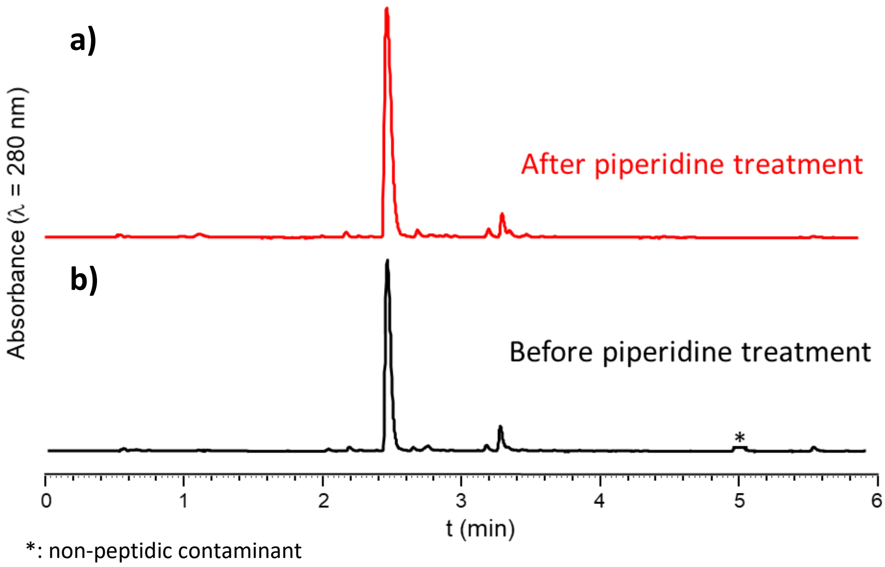

Supplementary figure S5: HPLC trace (Chromolith, gradient: 5-50% B/A over 5 min) of TFA-cleaved peptidyl resin **S5** with (a) and without (b) prior piperidine treatment

3-5- Optimization of the reaction of 2 with S3

- Table S2: Different conditions screened for the introduction of Ades

Entry	Equiv. 1	Base (20 equiv.)	Time (min)	S6 (%)[a]	S10 (%)[a]
1	10	DIEA	5	4	96
2	10	NMM	5	11	89
3	10	-	5	20	80
4	10	-	60	1	99
5	3	-	60	25	75
6	3 x 1.5	-	3 x 20	7	93

[a]: Relative rates determined by HPLC peak integration at λ= 280 nm taking into account the predicted molar absorption coefficient of Trp, disulfide [1] and *S*-Npys at 280 nm: ε_{Trp} = 5500 L.mol^{-1}.cm^{-1}; ε_{SS} = 125 L.mol^{-1}.cm^{-1}; ε_{Npys} = 1884 L.mol^{-1}.cm^{-1} (ε_{Npys} at 280 nm was determined from the molar absorption coefficient of 2,2'-dithiobis(5-nitropyridine) at 280 nm measured in 8:2:0.01 water/MeCN/TFA).

The use of 10 equiv. of 2-amino-1,1-dimethylethane-1-thiol hydrochloride **2** for 1 h without base (table S2, entry 4) gave satisfying results in terms of conversion and impurities rates.

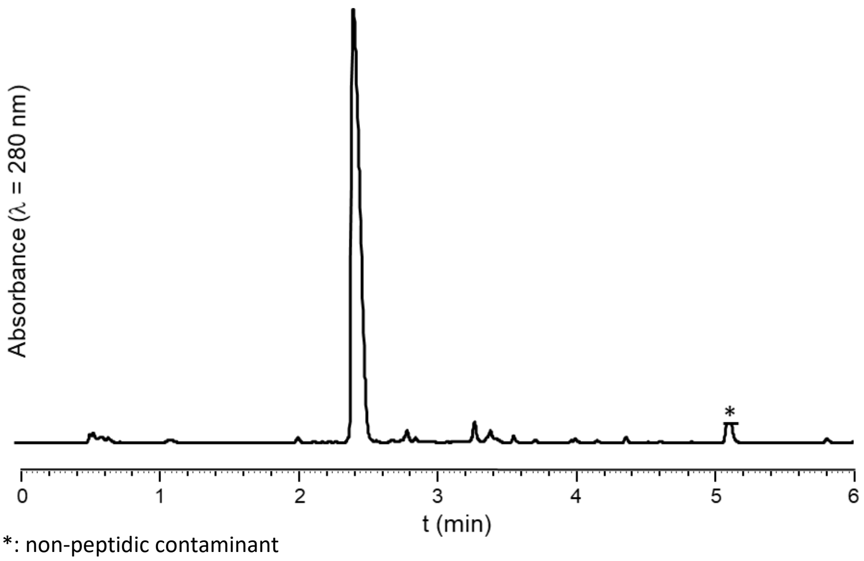

*: non-peptidic contaminant

Supplementary figure S6: HPLC trace (Chromolith, gradient: 5-50% B/A over 5 min) of crude **S10** obtained using optimized conditions (table S2, entry 4)

4- Synthesis and purification of MUC1 derived peptide segments

4-1- H-Trp-(MUC1)$_2$-(Hnb)Cys(S*t*Bu)-Gly-NH$_2$ crypto-thioester (3a)

Sequence: H-WAPDTRPAPGSTAPPAHGVTSAPDTRPAPGSTAPPAHGVTS-(Hnb)C(S*t*Bu)-G-NH$_2$

The peptide was synthesized following protocol PS1. Hnb was introduced following protocol PS2. Cleavage was performed following protocol PS4.

Elongation yield: 87%. Determined by the ratio between the quantity of fluorenylpiperidine released during final Fmoc deprotection (Protocol PS6) and the quantity released during the Fmoc deprotection of the C-terminal Gly residue (UV titration at 301 nm, ε = 7800 L.mol^{-1}.cm^{-1}).

ESI-MS (*m/z*): [M] calcd. for $C_{187}H_{284}N_{56}O_{60}S_2$: 4340.7, found: 4340.2 (average mass, deconvoluted)

HPLC purification: [5mg/mL] Nucleosil C18, gradient: 22-32% B/A over 20 min affording a white solid after lyophilisation (21% yield).

HPLC analysis: t_R = 4.32 min (Chromolith, gradient: 5-50% B/A over 5 min)

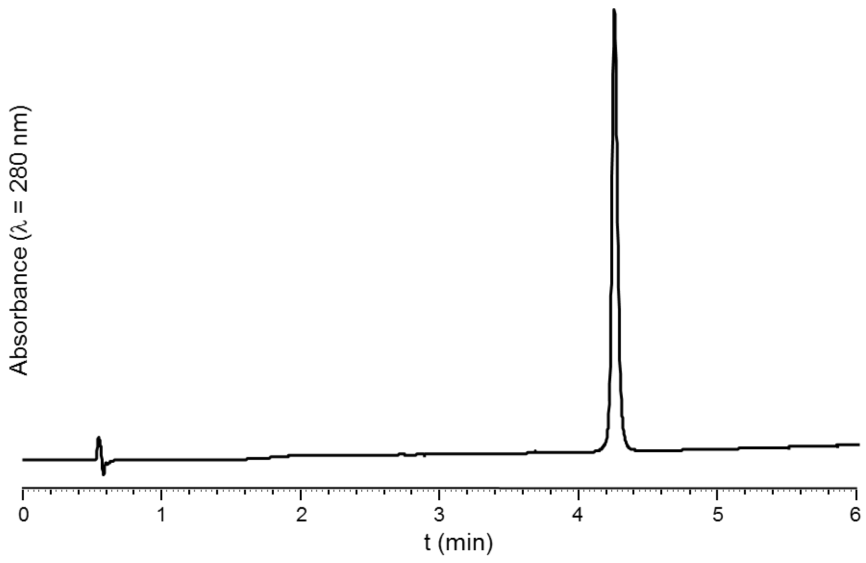

Supplementary figure S7: HPLC trace of purified compound **3a**

4-2 H-Cys(K_6-Ades)-(MUC1)$_2$-Trp-NH_2 cysteinyl peptide (7)

Sequence: H-C(H-KKKKKK-Ades)PDTRPAPGSTAPPAHGVTSAPDTRPAPGSTAPPAHGVTSW-NH_2

The peptide was synthesized following protocol PS1. Ades was introduced following protocol PS3. Cleavage was performed following protocol PS4.

Elongation yield: 86%. Determined by the ratio between the quantity of fluorenylpiperidine released during final Fmoc deprotection and the quantity released during the Fmoc deprotection of the C-terminal Trp residue (UV titration at 301 nm, ε = 7800 $L.mol^{-1}.cm^{-1}$).

ESI-MS (*m/z*): [M] calcd. for $C_{211}H_{345}N_{66}O_{61}S_2$: 4846.5, found: 4845.5 (average mass, deconvoluted)

HPLC purification: [5mg/mL] Nucleosil C18, gradient: 12-22% B/A over 20 min, affording a white solid after lyophilisation (22% yield).

HPLC analysis: t_R = 3.35 min (Chromolith, gradient: 5-50% B/A over 5 min)

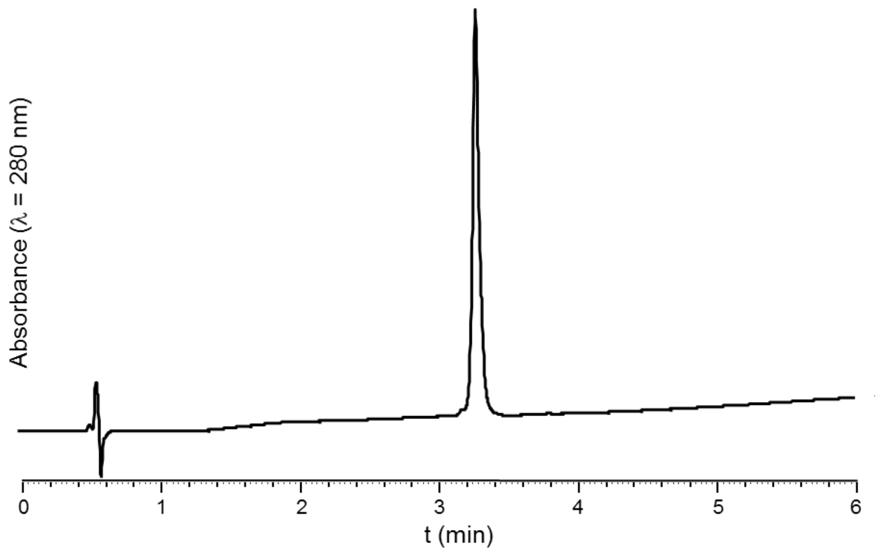

Supplementary figure S8: HPLC trace of purified compound **7**

4-3 H-Trp-(MUC1)$_2$-(Hnb)Cys(S*t*Bu)-Gly-(Lys)$_6$-NH$_2$ crypto-thioester (3b)

Sequence: H-WAPDTRPAPGSTAPPAHGVTSAPDTRPAPGSTAPPAHGVTS-(Hnb)C(S*t*Bu)-GKKKKKK-NH$_2$

The peptide was synthesized using protocol PS1. Hnb was introduced using protocol PS2. Cleavage was performed following protocol PS4.

Elongation yield: 90%. Determined by the ratio between the quantity of fluorenylpiperidine released during final Fmoc deprotection (Protocol PS6) and the quantity released during the Fmoc deprotection of the C-terminal Lys residue (UV titration at 301 nm, ε = 7800 L.mol-1.cm-1).

ESI-MS (*m/z*): [M] calcd. for $C_{223}H_{357}N_{71}O_{66}S_2$: 5110.8, found: 5110.3 (average mass, deconvoluted)

HPLC purification: [5mg/mL] Nucleosil C18, gradient: 22-32% B/A over 20 min affording a white solid after lyophilisation (22% yield).

HPLC analysis: t_R = 3.54 min (Chromolith, gradient: 5-50% B/A over 5 min)

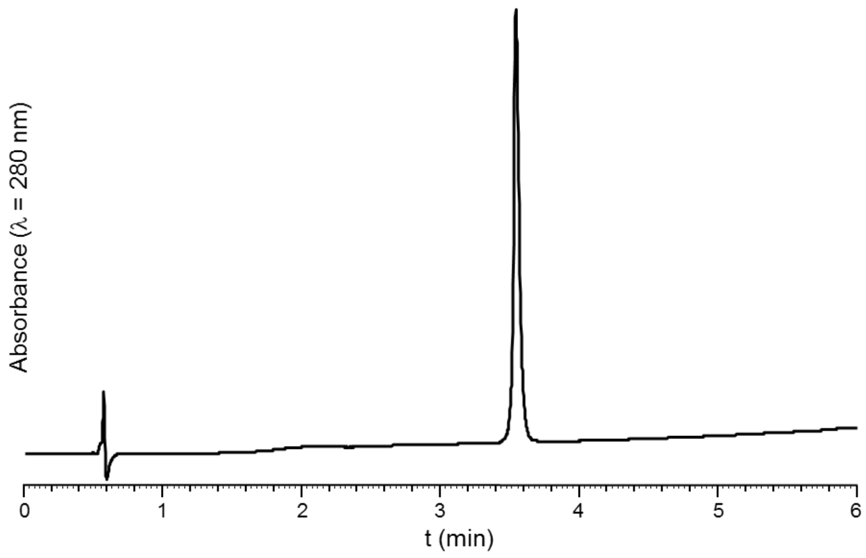

Supplementary figure S9: HPLC trace of purified compound **3b**

5- Synthesis and purification of SUMO-2 derived peptide segments

5-1 SUMO-2[1-47]-(Hnb)Cys(S*t*Bu)-Gly-NH$_2$ crypto-thioester (10)

Sequence: H-XADEKPKEGVKTENNDHINLKVAGQDGSVVQFKIKRHTPLSKLXKAY-(Hnb)C(S*t*Bu)-G-NH$_2$
(X= Norleucine)

The peptide was synthesized using protocol PS1. Hnb was introduced using protocol PS2. D26 and G27 were introduced through the coupling of dipeptide Fmoc-Asp(O*t*Bu)-(Dmb)Gly-OH to prevent aspartimide formation during SPPS (3.5 equiv. dipeptide, 3.4 equiv. HATU, 10 equiv. DIEA in NMP for 2 h). Cleavage was performed following protocol PS4.

Elongation yield: 90%. Determined by the ratio between the quantity of fluorenylpiperidine released during final Fmoc deprotection (Protocol PS6) and the quantity released during the Fmoc deprotection of the C-terminal Gly residue (UV titration at 301 nm, ε = 7800 L.mol^{-1}.cm^{-1}).

ESI-MS (*m/z*): [M] calcd. for $C_{250}H_{409}N_{71}O_{74}S_2$: 5657.5, found: 5657.6 (average mass, deconvoluted)

HPLC purification: [5mg/mL] Nucleosil C18, gradient: 25-40% B/A over 20 min affording a white solid after lyophilisation (37% yield).

HPLC analysis: t_R = 3.88 min (Chromolith, gradient: 5-50% B/A over 5 min)

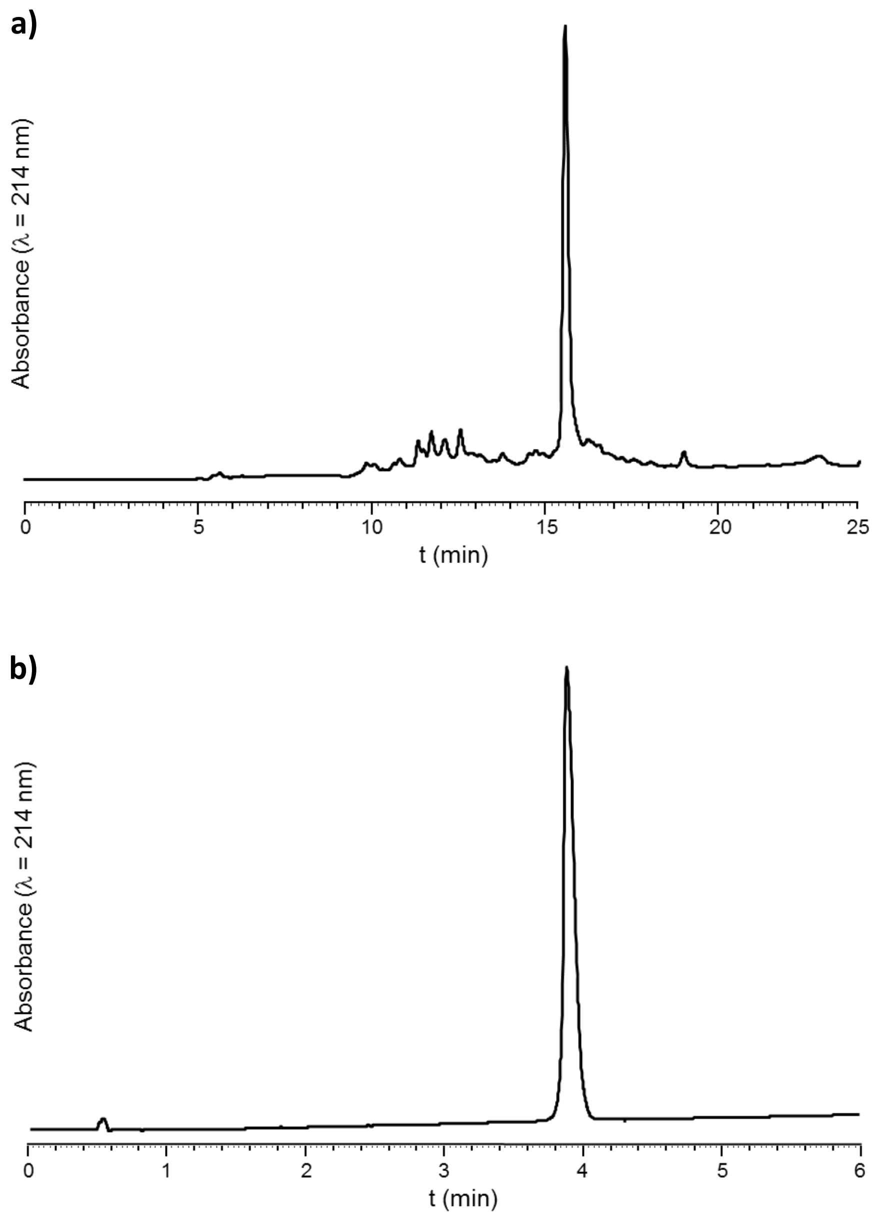

Supplementary figure S10: HPLC traces of crude (a) and purified (b) compound **10** (HPLC conditions: Nucleosil C18, gradient: 5-50% B/A over 40 min, and Chromolith, gradient 5-50% B/A over 5 min, respectively)

5-2- SUMO-2[48-93] cysteinyl peptide (S11)

Sequence: H-CERQGLSXRQIRFRFDGQPINETDTPAQLEXEDEDTIDVFQQQTGG-OH (X= Norleucine)

The peptide was synthesized following protocol PS1. D63 and G64 were introduced through the coupling of dipeptide Fmoc-Asp(O*t*Bu)-(Dmb)Gly-OH to prevent aspartimide formation during SPPS (3.5 equiv. dipeptide, 3.4 equiv. HATU, 10 equiv. DIEA in NMP for 2 h). Q88 and Q89 were subjected to double couplings. Cleavage was performed following protocol PS4.

Elongation yield: 79%. Determined by the ratio between the quantity of fluorenylpiperidine released during final Fmoc deprotection and the quantity released during the Fmoc deprotection of the C-terminal Gly residue (UV titration at 301 nm, ε = 7800 $L.mol^{-1}.cm^{-1}$).

Solubility: S11 was not soluble at 5 mg/mL and 1 mg/mL in 8:2:0.01 water/MeCN/TFA , which are the conditions used to purify the other segments. We were able to solubilize crude peptide at very low concentrations (0.056 mg/mL and 0.1 mg/mL in 8:2:0.01 and 6:4:0.01 water/MeCN/TFA, respectively). However, we observed a non-conventional HPLC behaviour, and dragging of the product leading to an overlap of the impurities and the desired product, in accordance with the finding of Brik and co-workers.[2]

ESI-MS (*m/z*): [M] calcd. for $C_{225}H_{354}N_{66}O_{80}S_1$: 5295.7, found: 5295.9 (average mass, deconvoluted)

HPLC Analysis: t_R = 17.2 min (Jupiter C4, gradient: 10-90% B/A over 20 min)

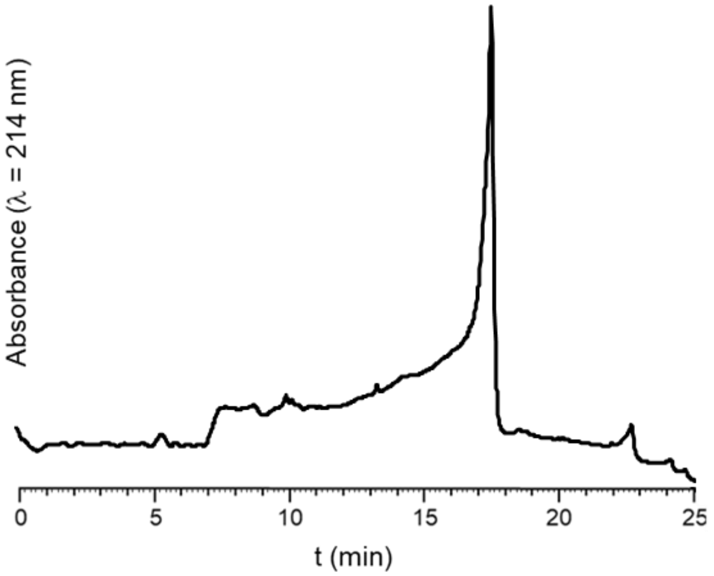

Supplementary figure S11: HPLC trace of crude peptide **S11**

5-3- Cys(K$_6$-Ades)-SUMO-2[49-93] cysteinyl peptide (12)

Sequence: H-C(H-KKKKKK-Ades)ERQGLSXRQIRFRFDGQPINETDTPAQLEXEDEDTIDVFQQQTGG-OH

(X= Norleucine)

The peptide was synthesized using protocol PS1- Peptide elongation. Ades was introduced following protocol PS3. D63 and G64 were introduced through the coupling of dipeptide Fmoc-Asp(O*t*Bu)-(Dmb)Gly-OH to prevent aspartimide formation during SPPS (3.5 equiv. dipeptide, 3.4 equiv. HATU, 10 equiv. DIEA in NMP for 2 h). Q88 and Q89 were subjected to double couplings. Cleavage was performed following protocol PS4. Boc-Cys(Ades) introduction was performed following the protocol PS3.

Elongation yield: 82%. Determined by the ratio between the quantity of fluorenylpiperidine released during final Fmoc deprotection and the quantity released during the Fmoc deprotection of the C-terminal Gly residue (UV titration at 301 nm, ε = 7800 $L.mol^{-1}.cm^{-1}$).

ESI-MS (*m/z*): [M] calcd. for $C_{265}H_{435}N_{79}O_{86}S_2$: 6167.9, found: 6166.8 (average mass, deconvoluted)

HPLC purification: [1 mg/mL] Jupiter C4, gradient: 7-42% B/A over 20 min affording a white solid after lyophilisation (14% yield).

HPLC analysis: t_R = 4.06 min (Chromolith, gradient: 5-50% B/A over 5 min)

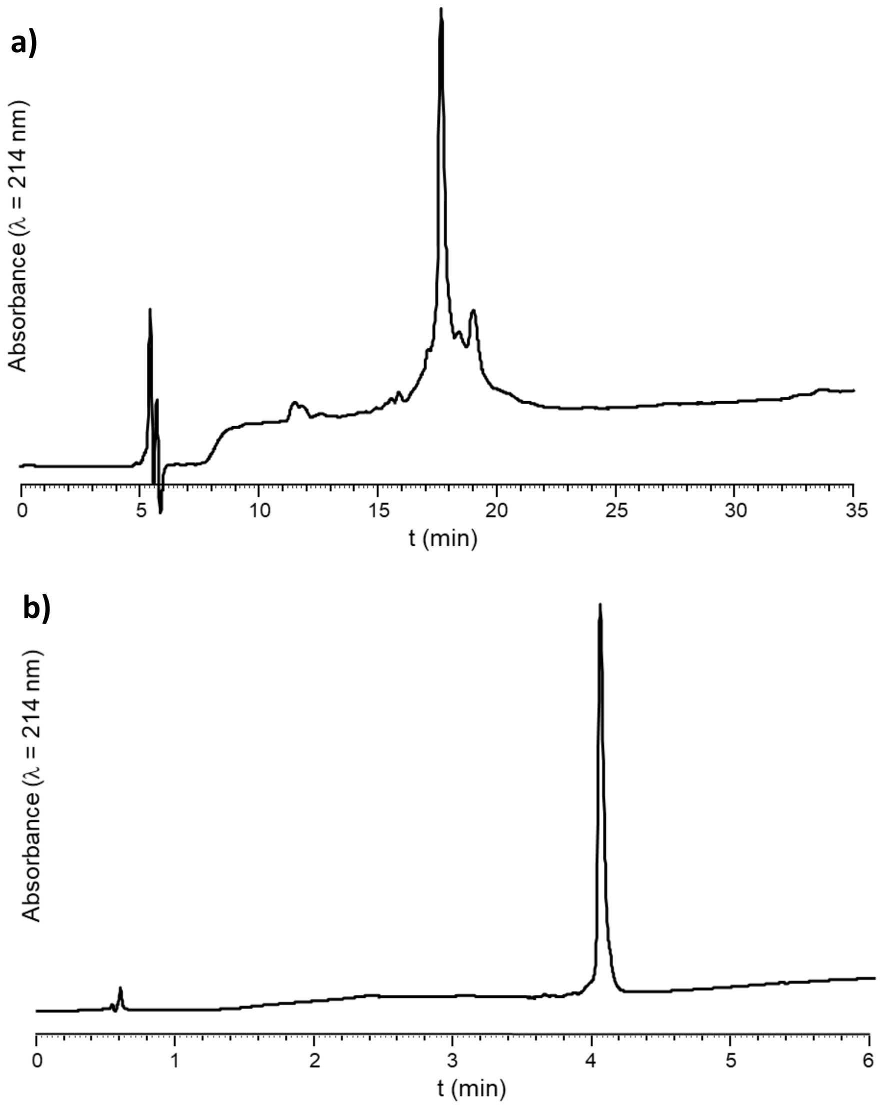

Supplementary figure S12: HPLC traces of crude (a) and purified (b) compound **12** (HPLC conditions: Nucleosil C18, gradient: 5-50% B/A over 40 min, and Chromolith, gradient 5-50% B/A over 5 min, respectively)

6- Native chemical ligations (NCL)

6-1 NCL between 3a and 7 to give 9

Crypto-thioester **3a** (3.0 mg, 0.6 µmol, 2 mM final concentration) and cysteinyl peptide **7** (5.4 mg, 0.9 µmol, 1.5 equiv., 3 mM final concentration) were dissolved in NCL buffer (300 µL) and the resulting yellow solution was gently stirred under inert atmosphere at 37°C for 12 h.

Note that ligation product **9** coeluted with *S*-deprotected cryptothioester **4a**. LC-MS analysis indicated total consumption of **4a** after 12 h reaction.

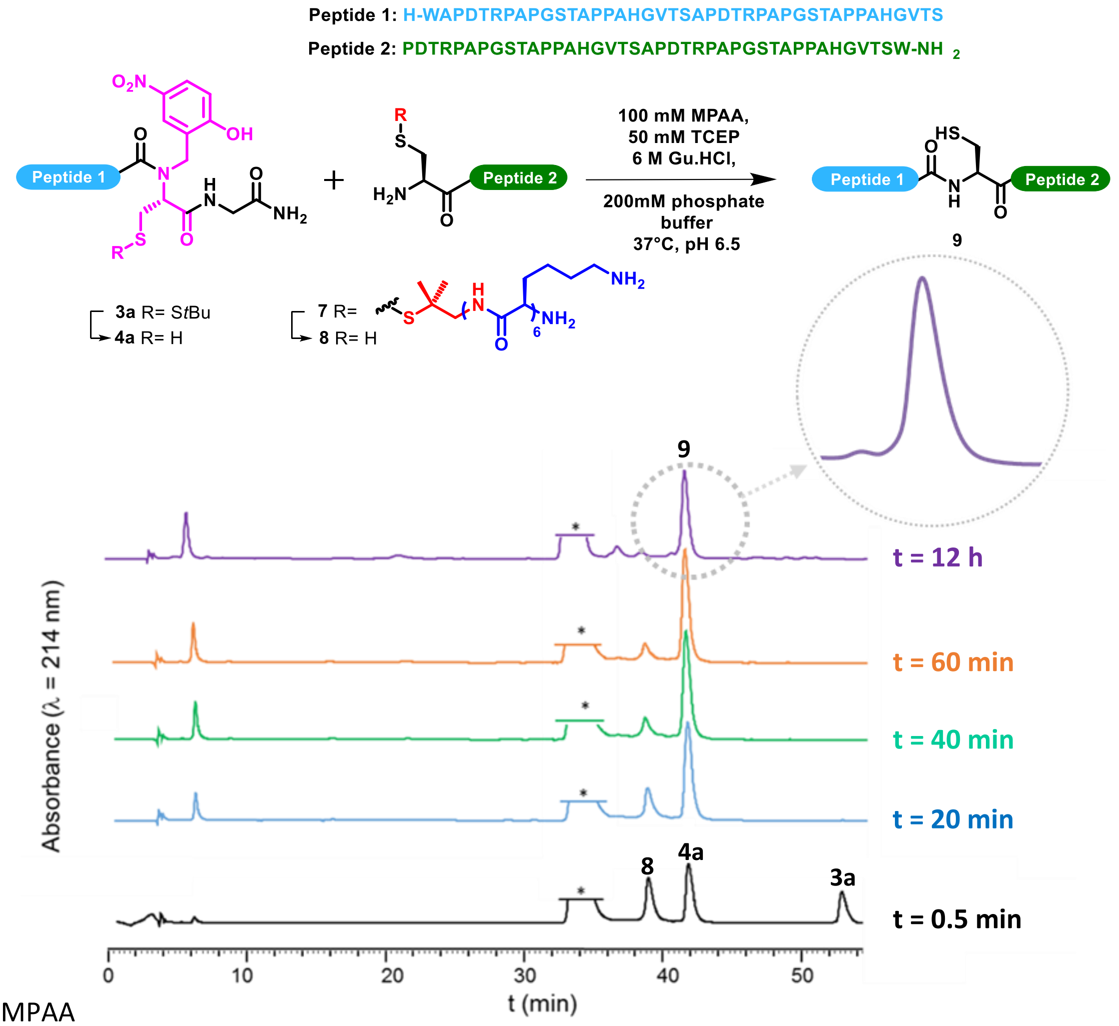

* = MPAA

Supplementary figure S13: HPLC (Nucleosil C18, gradient: 20-50% B/A over 60 min) monitoring of ligation between compounds **3a** and **7**

- Ligation product (**9**)

HPLC purification: [5mg/mL] Jupiter C4, gradient: 10-24% B/A over 20 min affording a white solid after lyophilisation (62% yield)

ESI-MS (*m/z*): [M] calcd. for $C_{342}H_{523}N_{105}O_{110}S_1$: 7897.5, found: 7896.7 (average mass, deconvoluted)

HPLC analysis: t_R = 3.38 min (Chromolith, gradient: 5-50% B/A over 5 min)

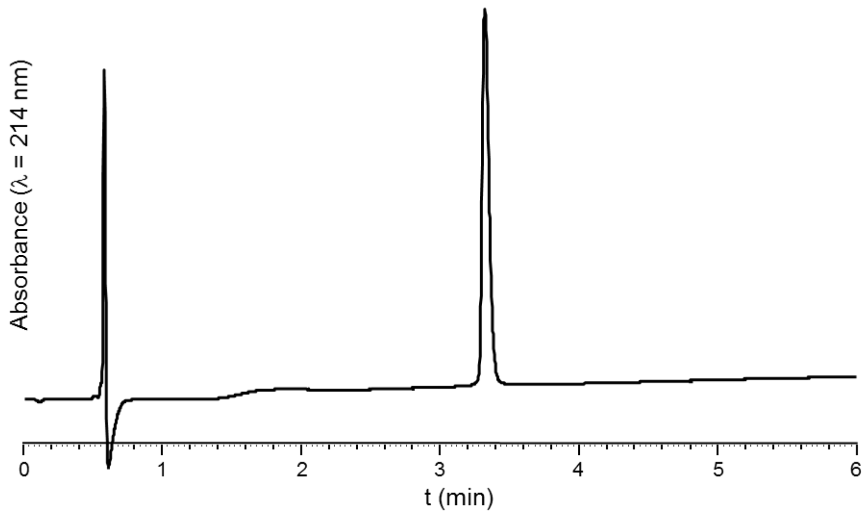

Supplementary figure S14: HPLC trace of purified ligation product **9**

6-2- NCL between 3b and 7 to give 9

Crypto-thioester **3b** (3.0 mg, 0.4 µmol, 2 mM final concentration) and cysteinyl peptide **7** (3.6 mg, 0.6 µmol, 1.5 equiv., 3 mM final concentration) were dissolved in NCL buffer (200 µL) and the resulting yellow solution was gently stirred under inert atmosphere at 37°C for 12 h.

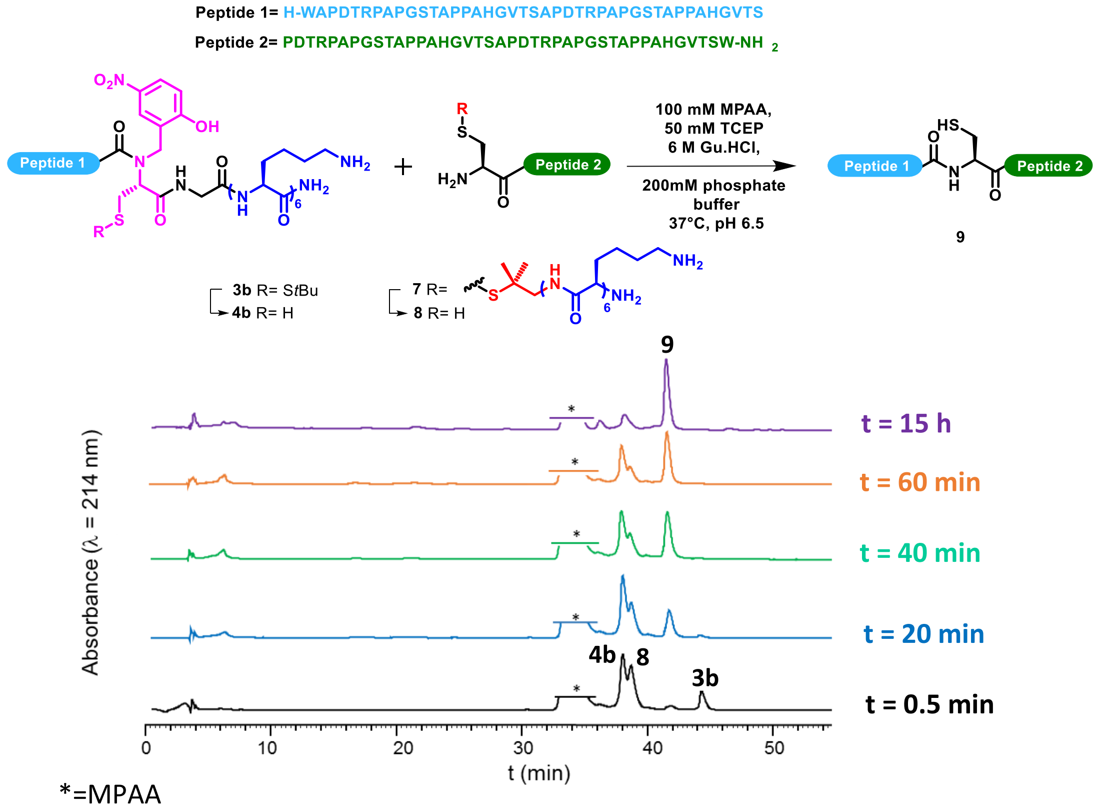

*=MPAA

Supplementary figure S15: HPLC (Nucleosil C18, gradient: 20-50% B/A over 60 min) monitoring of ligation between compounds **3b** and **7**

- Ligation product (**9**)

HPLC purification: [5mg/mL] Jupiter C4, gradient: 10-24% B/A over 20 min affording a white solid after lyophilisation (76% yield)

ESI-MS (*m/z*): [M] calcd. for $C_{342}H_{523}N_{105}O_{110}S_1$: 7897.5,found: 7896.7 (average mass, deconvoluted)

HPLC analysis: t_R = 3.38 min (Chromolith, gradient: 5-50% B/A over 5 min)

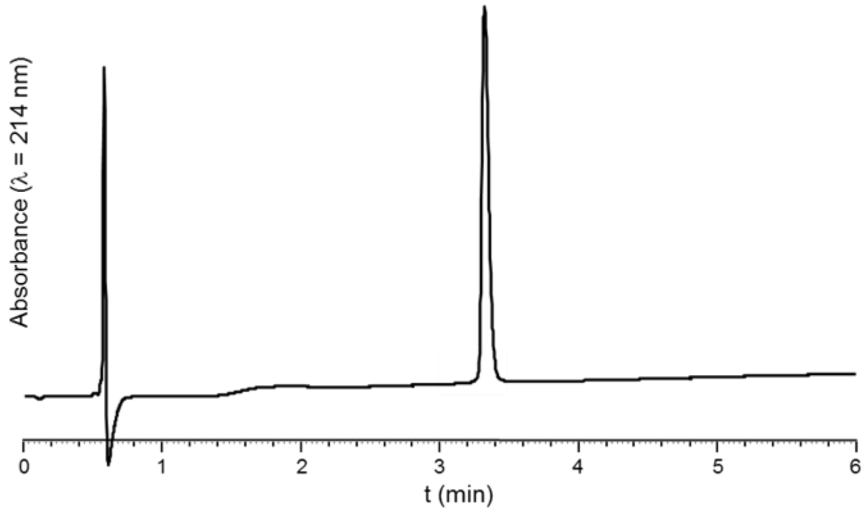

Supplementary figure S16: HPLC trace of ligation product **9** between compounds **3b** and **7**

6-3- Desulfurization of 9

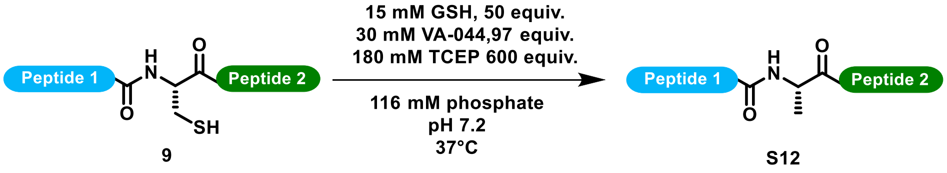

Cysteine-containing peptide **9** (0.55 mg, 61 nmol) was desulfurized by incubation for 12 h at 37°C in a 116 mM phosphate buffer (203 µL, 0.3 mM peptide) containing 15 mM reduced glutathione (GSH), 30 mM 2,2'-azobis[2-(2-imidazolin-2-yl)propane]dihydrochloride (VA-044) and 180 mM TCEP, pH = 7.2. LC-MS monitoring showed completion of the reaction. The crude product **S12** was not purified.

- Desulfurized product (**S12**)

ESI-MS (*m/z*): [M] calcd. for $C_{342}H_{523}N_{105}O_{110}$: 7865.5 average, found: 7865.7 (average mass, deconvoluted)

HPLC analysis: t_R = 6.14min (Aeris Widepore XB-C18 2, gradient: 3-90 % B'/A' over 15 min)

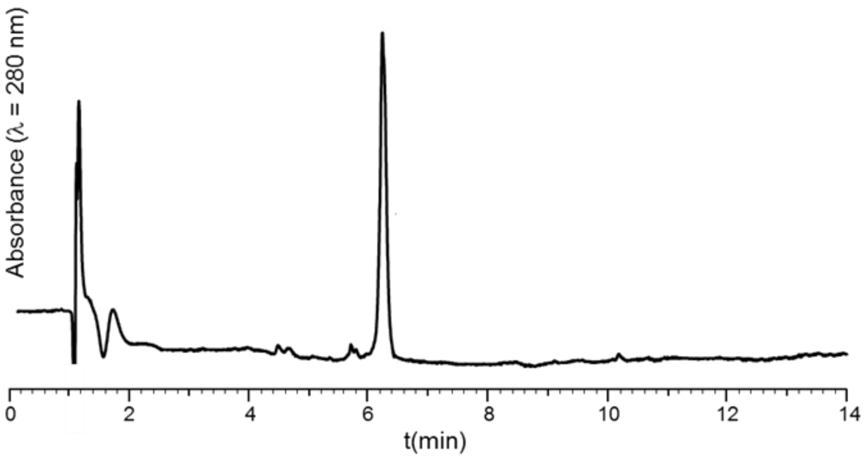

Supplementary figure S17: HPLC trace of crude desulfurized product **S12**

6-4 NCL between 10 and 12 to give SUMO-2[1-93] (14)

Crypto-thioester **10** (3.5 mg, 0.44 µmol, 2 mM final concentration) and cysteinyl peptide **12** (4.97 mg, 0.66 µmol, 1.5 equiv., 3 mM final concentration) were dissolved in NCL buffer (220 µL) and the resulting yellow solution was gently stirred under inert atmosphere at 25°C for 12 h.

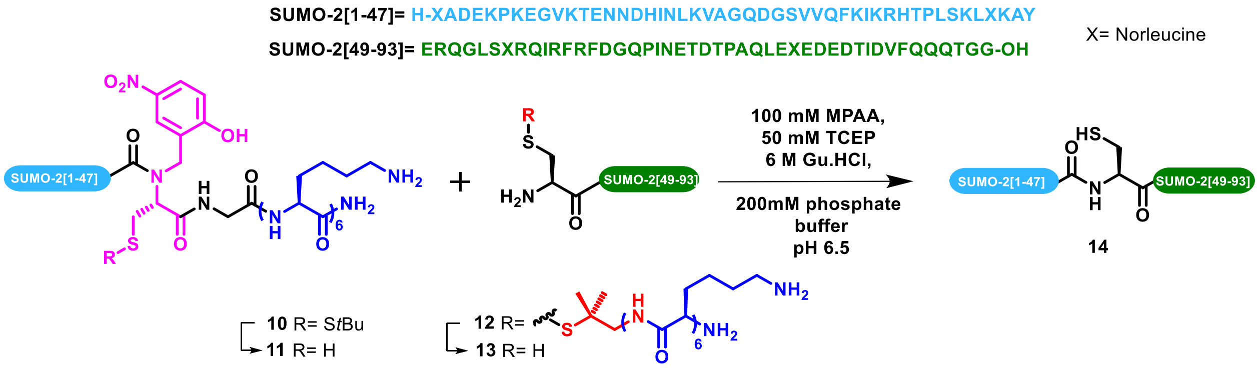

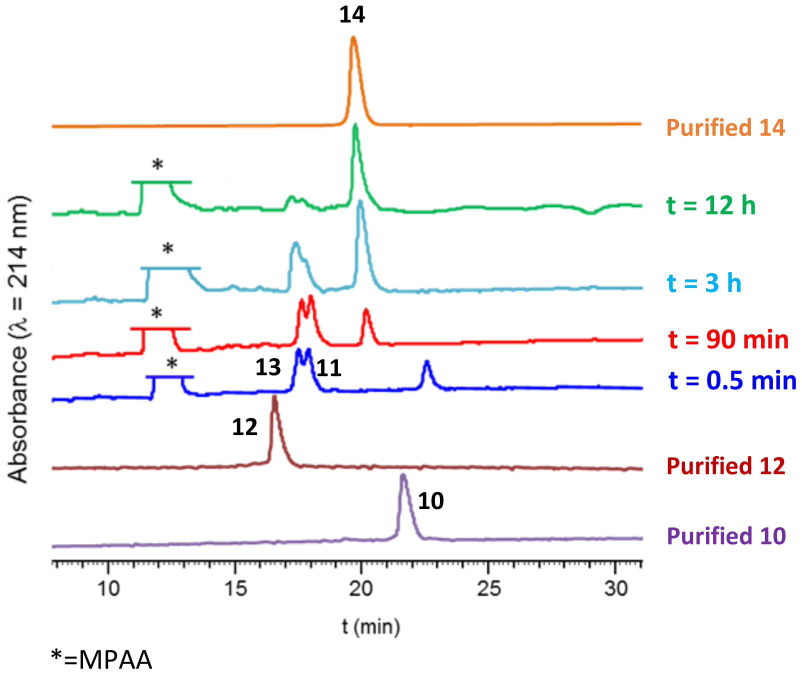

Supplementary figure S18: HPLC monitoring (Nucleosil C18, gradient: 20-50% B/A over 50 min) of ligation between compounds **10** and **12**

In accordance with the finding of Melnyk and co-workers[3] during the NCL-based synthesis of a SUMO-2 derivative using *bis*(2-sulfenylethyl)amido (SEA) crypto-thioesters, [4] a by-product **S13** which mass is consistent with aspartimide formation at one of the two Asp-Gly sites (-18 Da in MS analysis) was formed when conducing the reaction at 37°C for 12 h. The by-product **S13** co-eluted with SUMO-2 **14** in HPLC and LC-MS, and the relative rate (18% aspartimide) was quantified by integration of the extracted ion chromatograms corresponding to the expected product and aspartimide ($[M+12H]^{12+}$ peak, the most intense one of the multicharged envelopes) of the crude mixture. Similarly to Melnyk's findings, when the reaction was performed at 25°C for 12 h, no significant amount of the aspartimide by-product was formed (<5%).

- Ligation product SUMO-2[1-93] (**14**)

HPLC purification: [2mg/mL] Jupiter C4, gradient: 20-90% B/A over 40 min affording a white solid after lyophilisation (40% yield determined by weight, 36% (159 nmol) by UV spectrophotometry at λ = 280 nm, as compared to weighted initial amount of **10**)

ESI-MS (*m/z*): [M] calcd. for $C_{459}H_{739}N_{133}O_{149}S$: 10536.6, found: 10535.4 (average mass, deconvoluted)

ESI-HRMS (*m/z*): [M] calcd. for $C_{459}H_{739}N_{133}O_{149}S$: 10530.4059, found: 10530.4060 (deconvoluted)

HPLC analysis: t_R = 2.61 min (Chromolith, gradient: 20-50% B/A over 5 min)

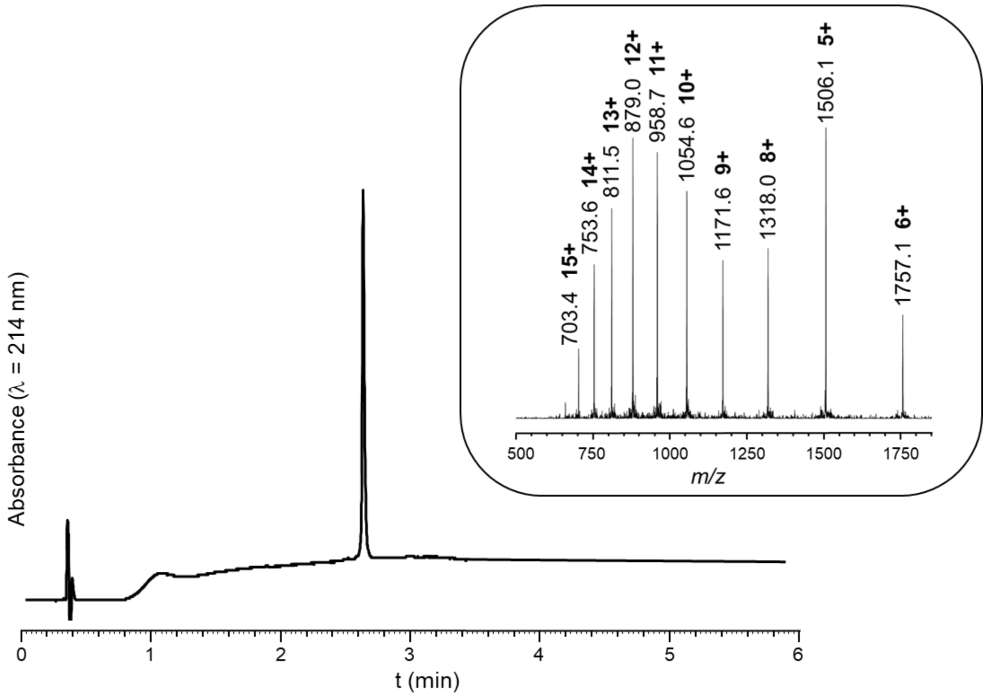

Supplementary figure S19: HPLC trace and ESI mass spectrum of purified SUMO-2[1-93] **14**

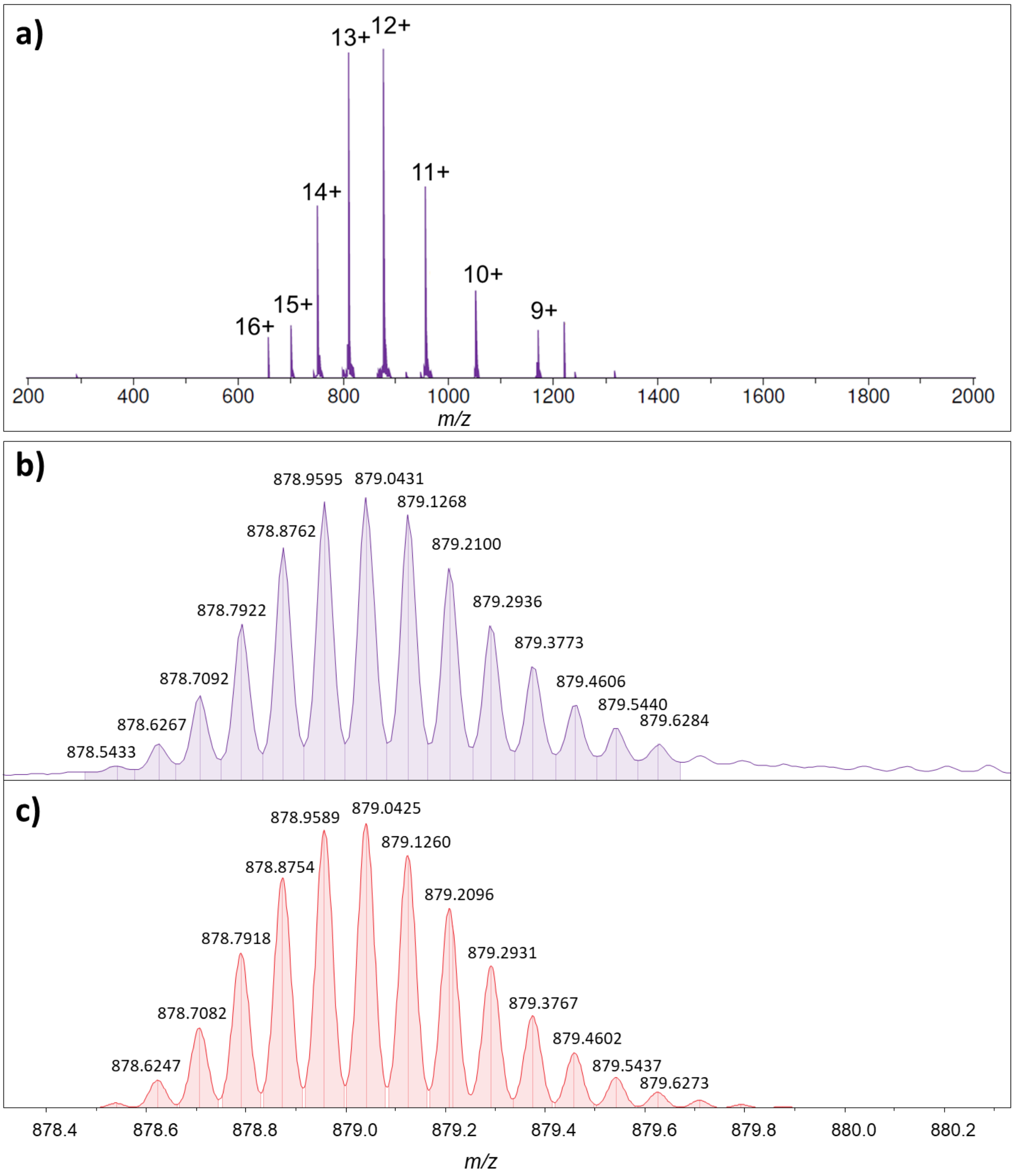

Supplementary figure S20: a) High resolution mass spectrum of purified SUMO-2[1-93] **14**; b) zoom on the $[M+12H]^{12+}$ peak; c) theoretical isotopic distribution of the $[M+12H]^{12+}$ peak

- Aspartimide by-product **S13**

Two possible sequences (since there are two aspartimide prone DG sites)**:**

H-XADEKPKEGVKTENNDHINLKVAGQDGSVVQFKIKRHTPLSKLXKAYCERQGLSXRQIRFRFZQPINETDTPAQLE XEDEDTIDVFQQQTGG-OH

H-XADEKPKEGVKTENNDHINLKVAGQZSVVQFKIKRHTPLSKLXKAYCERQGLSXRQIRFRFDGQPINETDTPAQLE XEDEDTIDVFQQQTGG-OH

X = norleucine **Z** =

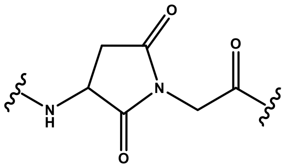

Note that we did not try to confirm the aspartimide structure nor to identify which of the two DG sequence could be more prompt for aspartimide formation during NCL.

ESI-MS (*m/z*): [M] calcd. for $C_{459}H_{737}N_{133}O_{148}S$: 10518.6, found: 10516.7 (average mass, deconvoluted)

HPLC analysis: t_R = 2.61 min (Chromolith, gradient: 20-50% B/A over 5 min)

7- Functional and structural characterization of SUMO-2[1-93] (14)

7-1- Circular dichroism

A 11.9 µM solution of SUMO-2 (**14**) in 10 mM sodium phosphate buffer (pH 7.2) containing 50 mM sodium fluoride was prepared and incubated at 25 °C for 12 h. Peptide concentration was determined by UV spectrophotometry at λ = 280 nm.[1]

The circular dichroism spectra were recorded on a Jasco 810 spectropolarimeter (J-810) at 25 °C over the range 190-260 nm using 0.1 cm path-length cell and by averaging 20 scans.
A 2 nm bandwidth, 1 nm data pitch were used for spectral acquisition and 2 s integration time.

The results are presented as $[\theta]_{MRW}$ against the wavelength between 190 and 260 nm.[5]

$$[\theta]_{MRW} = \frac{M \times \theta_{obs}}{(N\text{-}1) \times 10 \times d \times c}$$

with:

$[\theta]_{MRW}$ = mean residue ellipticity ($deg.cm^2.dmol^{-1}$)
M = molecular weight ($g.mol^{-1}$)
θ_{obs} = observed ellipticity (mdeg)
N = number of amino acids
d = pathlenght (cm)
c = concentration ($g.L^{-1}$)

The far-UV CD spectrum of **14** was compared to the one reported by Melnyk and his co-workers,[6] who analyzed [2-93]SUMO-2 at a ~10-22 µM concentration.
Spectra were almost identical in the 200-260 nm range.

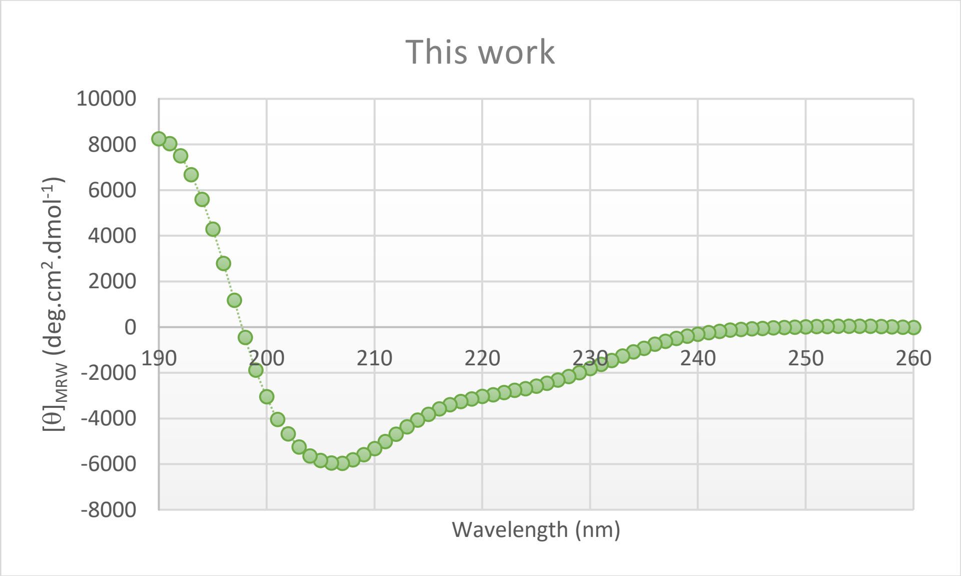

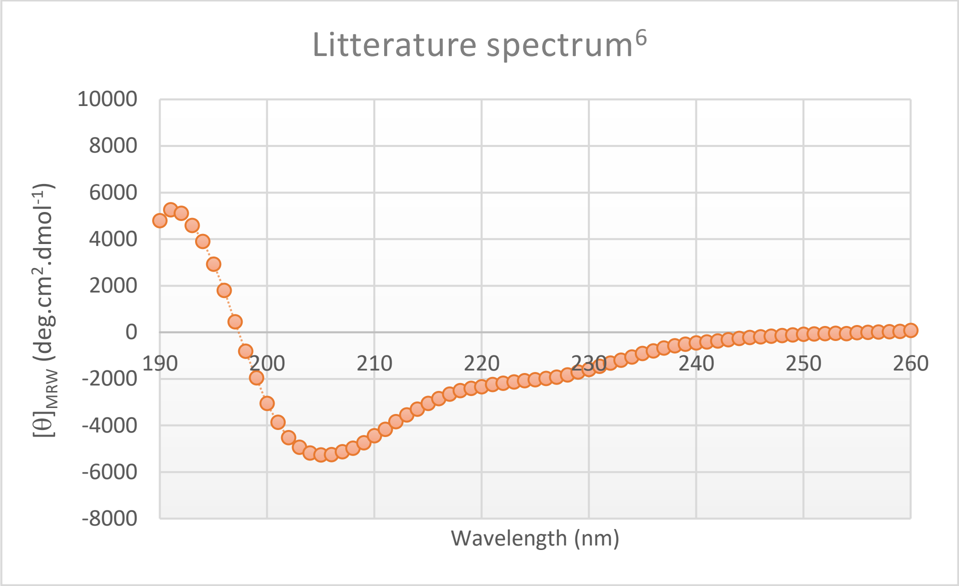

Supplementary figure S21: CD spectrum of **14** compared to a literature spectrum

7-2- Enzymatic conjugation

To perform enzymatic conjugation of synthetic SUMO-2, we used a SUMO-2 conjugation kit (Ref # K-715) developed by BostonBiochem containing SUMO E1 enzyme (SAE1/SAE2), SUMO E2 (Ubc9), recombinant SUMO-2, reaction buffer and a Mg^{2+}/ATP mix and we purchased the Ube2K (E2-25K) SUMOylation target from R & D System (Ref SP-200).

A solution of synthetic SUMO-2 (**14**) was prepared at the same concentration of recombinant SUMO-2 (250 μM) and in the same buffer (50 mM HEPES, 150 mM NaCl, 1 mM DTT, pH 7.5). SUMO conjugation reactions in the presence of synthetic or recombinant SUMO-2 were then performed according to the kit recommendations for 60 min. Mg^{2+}/ATP solution was omitted to obtain negative controls. Reaction was stopped by the addition of reducing SDS-PAGE sample buffer.

SUMO-2-conjugation of Ube2K was then analysed by western-blot (SDS-PAGE 15%) after heating the sample for 5 min at 90 °C. For this purpose, primary antibodies directed against SUMO-2/3 (Abcam, Ab1371) and Ube2K (Novus Biologicals, MAB6609) and secondary anti-mouse antibodies coupled to HRP (Invitrogen, Ref 61-6520) were used. The chemiluminescent HRP substrate Super signal west dura (Thermo Scientific, Ref 34075) allowed to reveal the corresponding proteins on a CL-Xposure film (Thermo Scientific, Ref 34090).

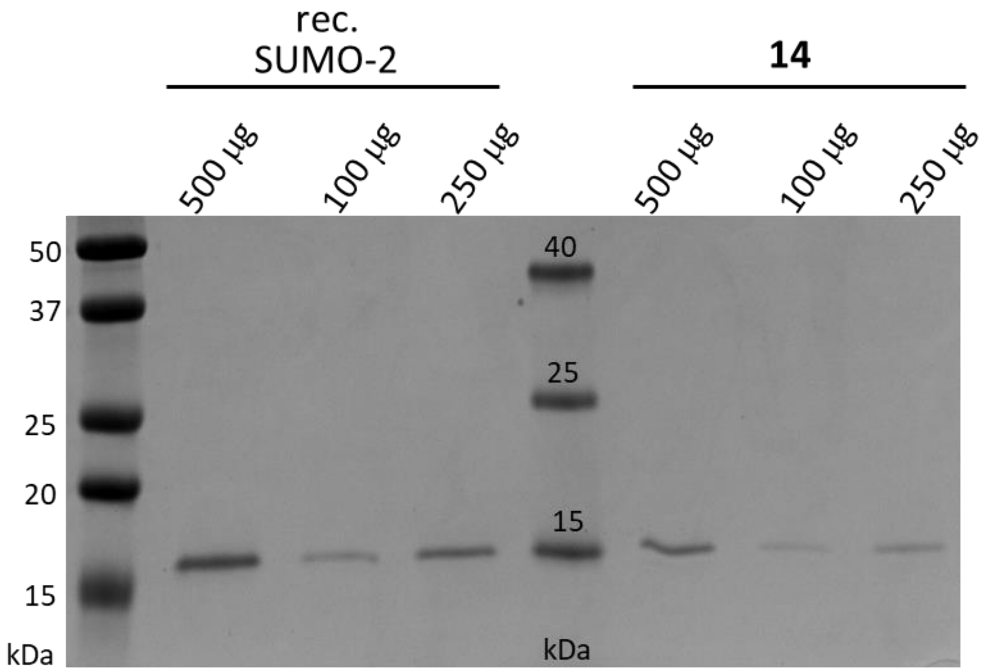

Supplementary figure S22: SDS-PAGE analysis of **14** and a recombinant SUMO-2 (coomassie blue staining)

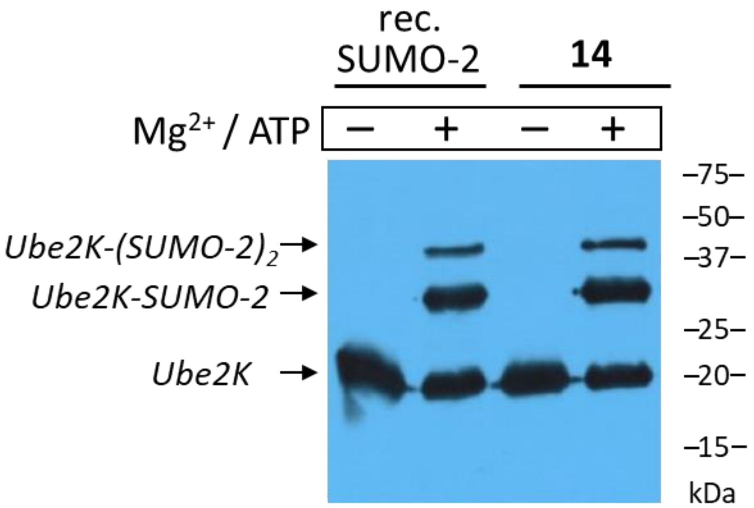

Supplementary figure S23: Anti-Ube2K western-blot of enzymatic SUMOylation of Ube2K by **14** and a recombinant SUMO-2

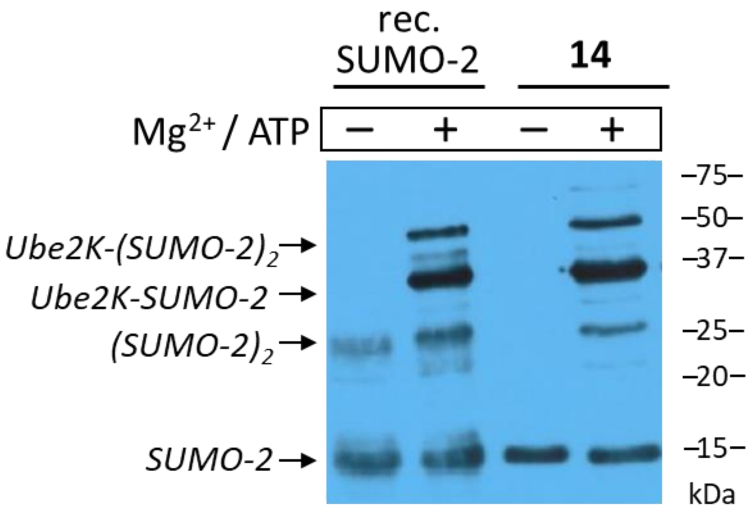

Supplementary figure S24: Anti-SUMO-2/3 western-blot of enzymatic SUMOylation of Ube2K by **14** and a recombinant SUMO-2

Another sample of synthetic SUMO-2 **14** was also denaturated in 6 M guanidium chloride and refolded[7] by diluting it ten times in reaction buffer (50 mM HEPES, 150 mM NaCl, 1 mM DTT, pH 7.5) such to obtain a final concentration of 250 μM before being tested as previously described in the enzymatic conjugation reaction. No difference was observed in the conjugation test between the sample simply diluted into buffer and the one subjected to this refolding protocol. Note that a different batch of conjugating kit was used for this experiment, explaining the lower Ube2K SUMOylation rates as compared to figure S24.

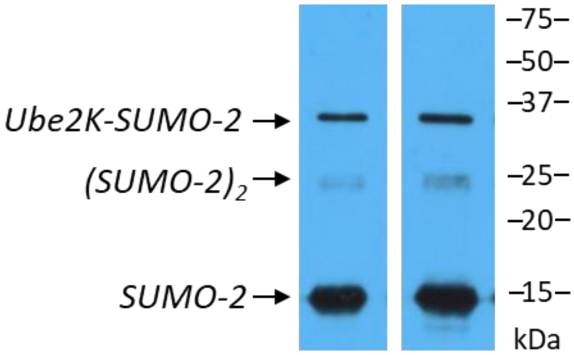

Supplementary figure S25: Anti-SUMO-2/3 western-blot of enzymatic SUMOylation of Ube2K by **14** either simply dissolved in buffer (left) or subjected to guanidinium chloride-mediated denaturation followed by refolding (right)

<u>**References**</u>:

1 C. N. Pace, F. Vajdos, L. Fee, G. Grimsley and T. Gray, How to measure and predict the molar absorption coefficient of a protein, *Protein Sci.*, 1995, **4**, 2411–2423.

2 S. Bondalapati, E. Eid, S. M. Mali, C. Wolberger and A. Brik, Total chemical synthesis of SUMO-2-Lys63-linked diubiquitin hybrid chains assisted by removable solubilizing tags, *Chem. Sci.*, 2017, **8**, 4027–4034.

3 J. Bouchenna, M. Sénéchal, H. Drobecq, J. Vicogne and O. Melnyk, The Problem of Aspartimide Formation During Protein Chemical Synthesis Using SEA-Mediated Ligation, 2019, In: Iranzo O., Roque A. (eds) Peptide and Protein Engineering. Springer Protocols Handbooks. Humana, New York, NY.

4 N. Ollivier, J. Dheur, R. Mhidia, A. Blanpain and O. Melnyk, Bis(2-sulfanylethyl)amino Native Peptide Ligation, *Org. Lett.*, 2010, **12**, 5238–5241.

5 N. Greenfield and G. D. Fasman, Computed circular dichroism spectra for the evaluation of protein conformation, *Biochemistry*, 1969, **8**, 4108–4116.

6 M. Sénéchal, J. Bouchenna, J. Vicogne and O. Melnyk, Comment on "N-terminal Protein Tail Acts as Aggregation Protective Entropic Bristles: The SUMO Case", *Biomacromolecules*, 2020, **21**, 3480–3482.

7 For a recent study on SUMO-2 refolding after denaturation, see: T. Nandi, A. Yadav, S. R. Koti Ainavarapu, Experimental comparison of energy landscape features of ubiquitin family proteins, *Proteins*, 2019, **88**, 449–461.
